# Identification and localization of polar tube proteins in the extruded polar tube of the microsporidian *Anncaliia algerae*

**DOI:** 10.1038/s41598-023-35511-y

**Published:** 2023-05-30

**Authors:** Maurine Fayet, Nastasia Prybylski, Marie-Laure Collin, Eric Peyretaillade, Ivan Wawrzyniak, Abdel Belkorchia, Reginald Florian Akossi, Marie Diogon, Hicham El Alaoui, Valérie Polonais, Frédéric Delbac

**Affiliations:** “Laboratoire “Microorganismes: Génome et Environnement”, CNRS, Université Clermont Auvergne, 63000 Clermont-Ferrand, France

**Keywords:** Cell biology, Microbiology, Pathogenesis

## Abstract

Microsporidia are obligate intracellular parasites able to infect a wide range of hosts from invertebrates to vertebrates. The success of their invasion process is based on an original organelle, the polar tube, which is suddenly extruded from the spore to inoculate the sporoplasm into the host cytoplasm. The polar tube is mainly composed of proteins named polar tube proteins (PTPs). A comparative analysis allowed us to identify genes coding for 5 PTPs (PTP1 to PTP5) in the genome of the microsporidian *Anncaliia algerae*. While PTP1 and PTP2 are found on the whole polar tube, PTP3 is present in a large part of the extruded polar tube except at its end-terminal part. On the contrary, PTP4 is specifically detected at the end-terminal part of the polar tube. To complete PTPs repertoire, sequential sporal protein extractions were done with high concentration of reducing agents. In addition, a method to purify polar tubes was developed. Mass spectrometry analysis conducted on both samples led to the identification of a PTP3-like protein (PTP3b), and a new PTP (PTP7) only found at the extremity of the polar tube. The specific localization of PTPs asks the question of their roles in cell invasion processes used by *A. algerae*.

## Introduction

Microsporidia are obligate intracellular parasites related to fungi. There are about 220 genera and 1700 species which are able to infect a wide range of hosts from insects to mammals, including humans^1^. The interest in microsporidia has increased in recent decades due to their involvement in human disorders predominantly in immunocompromised patients including people with HIV and people who take immunosuppressive drugs^2,3^. However, microsporidia are not only considered as opportunistic pathogens as they can also be the cause of infections in immunocompetent hosts^4,5^. Several species can infect humans including *Anncaliia* (syn. *Nosema*, *Brachiola*) *algerae* originally identified in *Anopheles stephensi* mosquitoes^6^. This species is considered as an emerged cause of myositis but was also associated with vocal cord, skin, ocular or disseminated infections mainly in immunosuppressed patients^7^. *A. algerae* is also known to develop infections in SCID mice^8^ and was more recently shown to infect *Drosophila melanogaster*^9^. In vitro, this microsporidian species can be grown in a wide range of cell lines including insect^10^, fish^11^ and mammalian cells^12^.

Microsporidia are characterized by an infectious and highly resistant form in the environment, the spore, which varies in size from approximately 1 to 20 µm depending on the species. The spore is surrounded by a thick and rigid cell wall which delineates the sporoplasm i.e. the infectious content of the parasite^13^. Microsporidia also receive a great attention because of their unique organelle named the polar tube which is involved in one of the most fascinating invasion mechanisms. When spores are exposed to not well defined environmental conditions, this highly specialized coiled structure is rapidly extruded and forms a hollow tube that is required for the transport and the delivering of the sporoplasm (including the nucleus) into the host cell cytoplasm^14^. This process called spore germination occurs in less than 2 s.

The polar tube is a multiprotein complex known to resist dissociation in detergents and acids but is solubilized with reducing agents such as dithiothreitol (DTT) or 2-mercaptoethanol (2-ME), suggesting that disulfide bridges play a major role in stabilizing this structure^15^. Studies conducted so far on its composition have resulted in the identification of core proteins known as polar tube proteins (PTPs). Six PTPs (PTP1 to PTP6) were described in numerous microsporidian species^15,16^. PTP1, the first polar tube component identified in both *Encephalitozoon cuniculi* and *E. hellem*^17,18^, is an acidic proline-rich protein. In addition, this PTP is O-mannosylated and is characterized by a high degree of sequence divergence between species^19,20^. PTP2 proteins are lysine-rich proteins and have a basic isoelectric point. Both PTP1 and PTP2 present many cysteine residues which could be involved in intra- and/or inter-protein disulfide bridges. It was demonstrated that *Encephalitozoon* spp *ptp1* and *ptp2* genes are organized in cluster and this organization seems to be conserved between several microsporidian genomes^21,22^. PTP3, firstly described in *E. cuniculi* by immunoscreening of a cDNA library^23^, is a higher molecular weight protein (> 1100 aa) rich in charged amino acids and, unlike both PTP1 and PTP2, is soluble in the absence of thiol-reducing agent. Proteins from the PTP3 family are found in all available microsporidian proteomes and were shown to be more conserved than PTP1 and PTP2. Interestingly, both cross-linked polar tube protein extracts and yeast two hybrid assays revealed that PTP1, PTP2 and PTP3 interact with each other^23,24^. Devoid of cysteine, PTP3 might be a scaffold protein by interaction with other PTPs. Thereafter, a proteomic-based approach in *E. cuniculi* allowed the identification of two novel PTPs named PTP4 and PTP5^25^. The *E. cuniculi* PTP4 protein is rich in glutamate, whereas PTP5 is rich in lysine residues and presents 20% of similarities with the PTP4 sequence^26^. Like the *ptp1* and *ptp2* genes, the *ptp4* and *ptp5* genes are also found in cluster and orthologs are present in many microsporidian genomes^15^. The more recently identified PTP6, described in the silkworm parasite *Nosema bombycis*, is a histidine and serine-rich protein whose orthologs are identified in only a limited number of species (*Encephalitozoon* spp and *N. ceranae*, a honeybee parasite) and able to bind to the host cell surface^16^. Because of the high evolution rates in microsporidia, PTPs are characterized by a high degree of sequence divergences between species making the identification of orthologs difficult.

Finally, studies highlighted the role of some PTPs in invasion processes through interactions with host cell surface. PTP1 via its O-linked mannosylation sites likely interact with host cell mannose-binding receptors facilitating adherence of the polar tube to the host cell membrane^19,20^. More recently, a unique epitope of the *E. hellem* PTP4 specifically localized at the tip of the extruded polar tube was shown to bind to the host transferrin receptor 1. This interaction was demonstrated to play a key role in microsporidian invasion^27^.

The objective of our study was to characterize the diversity of PTPs in *A. algerae* using different complementary approaches including homology search and differential sporal protein extractions with high concentrations of thiol-reducing agents associated with antibody-based approaches. We also developed a protocol to purify extruded polar tubes combined with mass spectrometry to identify potential new polar tube components. These strategies allowed us to identify 7 PTPs with distinct localization in the extruded polar tubes. Interestingly, PTP3 was shown to be absent at the tip of the extruded polar tubes whereas both PTP4 and the newly identified PTP7 only stained the terminal end of the polar tubes, suggesting a role of these proteins in binding to host cell receptor(s) during invasion process.

## Results

### Identification of genes coding for polar tube proteins PTP1 to PTP5 in *A*. *algerae*

A tBLASTn search against the *A. algerae* genome using PTP sequences from different microsporidian species allowed the identification of CDSs that share homologies with the five previously described PTP families. Complete sequences of genes encoding PTP1 (407 aa, WAQ68434), PTP3 (1203 aa, WAQ68436), PTP4 (254 aa, WAQ68438) and PTP5 (240 aa, WAQ68439) were identified (Table 1). PTP1 is characterized by a high proline content (32%) and an acidic isoelectric point. This protein contains 13 cysteine residues and a large number of potential O-glycosylation sites. PTP3 is a high molecular weight protein (127 kDa) with an acidic pI and a richness in both alanine and glutamate residues. This PTP contains only two cysteine residues and similarly to PTP1 is characterized by the presence of numerous potential O-glycosylation sites. Both PTP4 and PTP5 proteins are smaller lysine-rich proteins with similar molecular weights of 28 kDa. Three partial sequences coding for PTP2-like proteins were found in the genome assembly data of *A. algerae*. They vary from 418 to 492 aa in length and lack the N-terminus part, suggesting the presence of different PTP2 encoding genes or allelic variants (see Supplementary Fig S1). A 5’-RACE-PCR approach allowed the identification of the full-length sequence of the PTP2c protein. PTP2c complete protein (535 aa, WAQ68435) has a basic isoelectric point with a serine- and glycine-rich N-terminal region. Sequence alignment of the three PTP2-like proteins reveals that they are characterized by a highly conserved lysine-rich C-terminal region and a variable N-terminal part containing a large number of serine and glycine-rich repeated motifs (see Supplementary Fig S1). These sequences also contain 8 cysteine residues in conserved positions in their C-terminal part.

**Table 1:** Major characteristics of the 7 polar tube proteins (PTPs) identified in *Anncaliia* *algerae*.

PTP	Accession number	Complete protein	Mature protein	Isoelectric point (pI)	Major amino acid (%)	Number of cysteine residues	Number of potential O-glycosylation sites
PTP1	WAQ68434	407 aa	387 aa (39 kDa)	4.6	Pro (32%)	13	57
PTP2*	WAQ68435	535 aa	517 aa (49 kDa)	9.0	Gly (25%)	8	93
PTP3	WAQ68436	1203 aa	1183 aa (127 kDa)	5.6	Ala (15%)	2	54
PTP3b	WAQ68437	1197 aa	1182 aa (121 kDa)	5.0	Ala (16%)	5	84
PTP4	WAQ68438	254 aa	241 aa (28 kDa)	6.8	Lys (12%)	5	4
PTP5	WAQ68439	240 aa	240 aa (28 kDa)	8.9	Lys (14%)	8	1
PTP7	WAQ68440	417 aa	402 aa (45.5 kDa)	6.8	Thr (11%)	6	23

The mature proteins correspond to polypeptides devoid of their predicted N-terminal signal peptide. The pI, major amino acid and numbers of both cysteine residues and potential O-glycosylation sites are deduced from the mature proteins.

*PTP* polar tube protein, *aa* amino acids, *kDa* kilodalton.

*Only the data of a full length PTP2 sequence (PTP2c, see Supplementary Fig S1) are presented.

### Differential localization of *A*. *algerae* PTPs in the extruded polar tubes

**Figure 1:**
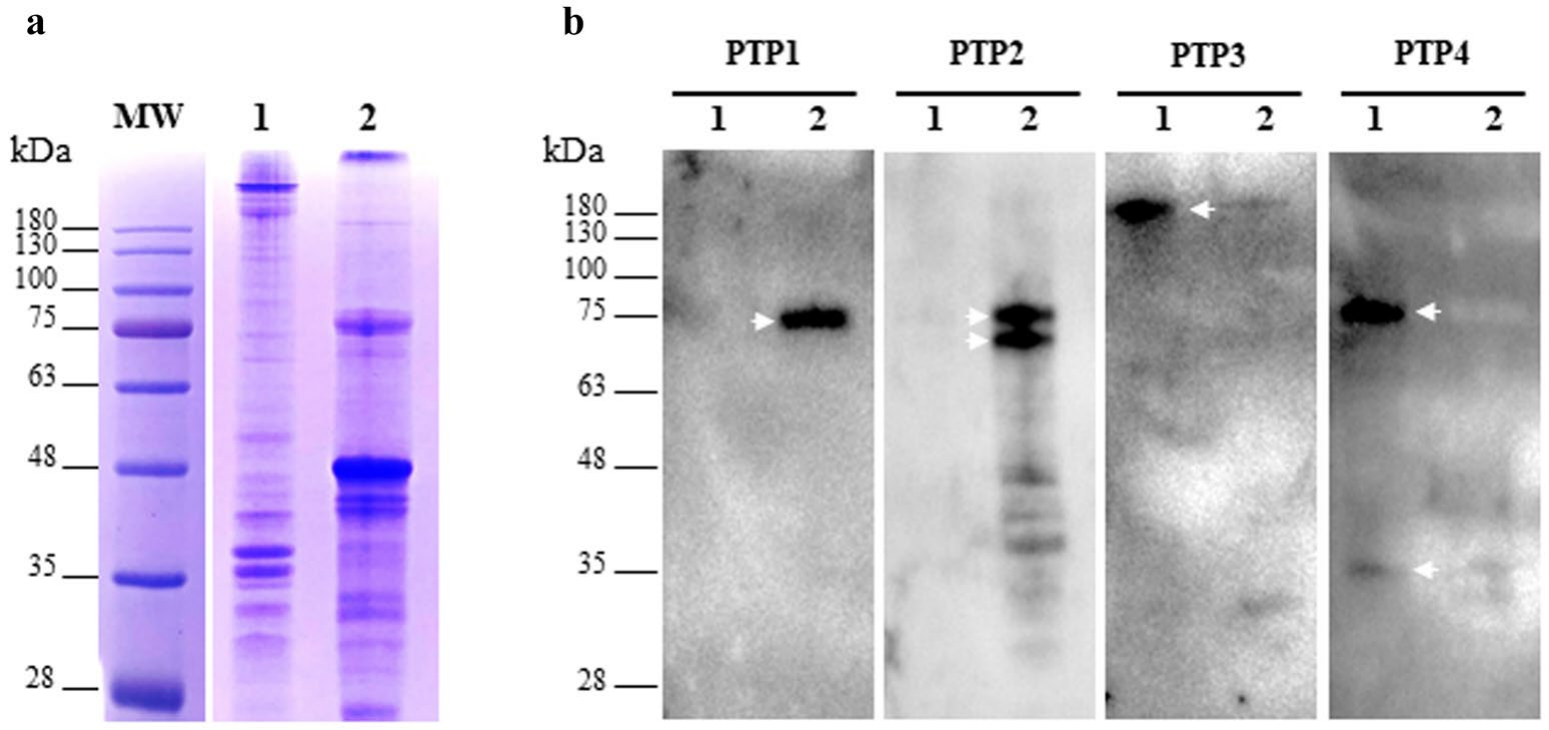
Immunodetection of PTPs in *A. algerae* sporal protein extracts. (**a**) Coomassie blue stained 10% SDS-PAGE of proteins extracted from *A. algerae* spores. The first extraction was done in 2.5% SDS (lane 1). Then, the insoluble proteins were solubilized in a buffer containing 100 mM DTT (lane 2). (**b**) Western Blot analysis using antisera raised against PTP1, PTP2, PTP3 and PTP4 on SDS (lane 1) and DTT extracts (lane 2). PTP1 and PTP2 are only soluble in presence of reducing agent (arrows). In contrast, PTP3 and PTP4 are detected in the SDS extract (arrows). All antisera were used at a 1:200 dilution.

**Figure 2:**
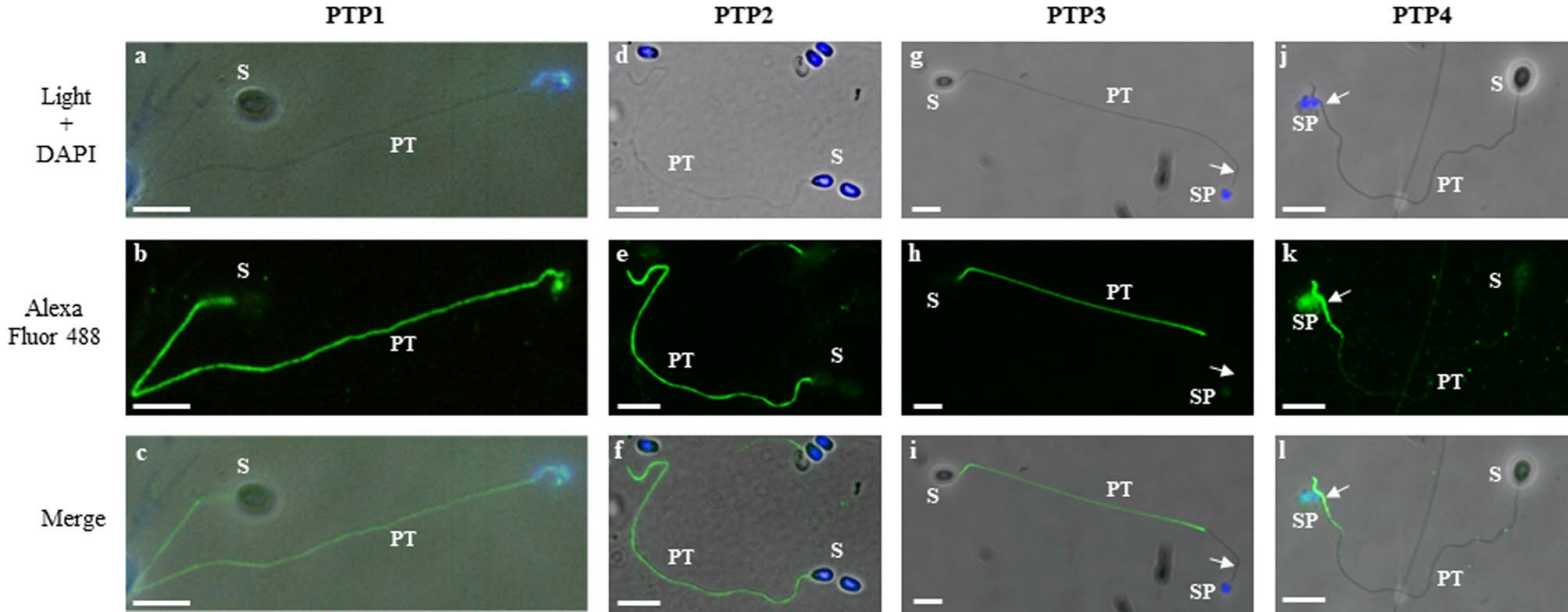
Indirect immunofluorescence assay using antisera raised against recombinant PTPs revealing the specific location of each PTP in *A. algerae* extruded polar tubes. Germinated spores are visualized in phase contrast (**a**,**d**,**g**,**j**), and after anti-PTP1 (**b**), anti-PTP2 (**e**), anti-PTP3 (**h**) and anti-PTP4 (**k**) labelling. Merged pictures were generated with GIMP 2.10.8 software (**c**,**f**,**i**,**l**). Antibodies against PTP1 and PTP2 proteins labelled the entire polar tube. In contrast, antibodies against the PTP3 protein labelled a large part of the polar tube except the extremity, whereas anti-PTP4 antibodies exclusively labelled the extremity of the polar tube (arrows). All antisera were used at a 1:100 dilution. Secondary antibody was Alexa 488-conjugated goat anti-mouse (or anti-rabbit for PTP1) IgG. At least 10 different fields were observed which corresponds to ~ 50 to 130 spores with their polar tube extruded. See Supplementary Fig. S2 for additional pictures illustrating the different localizations of PTPs. *PT* polar tube, *S* spore, *SP* sporoplasm. Scale bar: 10 µm.

Recombinant partial PTPs (PTP2, PTP3, PTP4 and PTP5) were produced in *E. coli*, affinity-purified and injected in mice in order to produce specific antibodies. Unfortunately, we did not succeed in expressing the whole PTP1 in *E. coli*, and attempts to express only the N-terminal part or the C-terminal part of PTP1 were also unsuccessful (see Supplementary Table S1). Anti-PTP1 antibodies were thus produced in rabbits against two synthetic peptides of the protein. The different antisera were then used to detect each PTP both in *A. algerae* protein extracts and in IFA. Antibodies to PTP1 peptides specifically recognized a unique protein band of 75 kDa in size (Fig. 1). This band, higher than the expected size, was only detected when sporal proteins were solubilized in presence of DTT. Similarly, mouse antibodies raised against the recombinant PTP2 reacted with cell lysates of *A. algerae* spores only in the DTT extract. These antibodies consistently recognized two closely running protein bands at ~ 70 and 75 kDa (Fig. 1). In contrast, a protein band around 180-kDa was stained with anti-PTP3 antibodies in protein extracts without any reducing agent. Finally, PTP4 antisera also recognized proteins in the SDS extract, with two main bands at 75 and 35 kDa. Unfortunately, antisera raised against PTP5 did not detect any protein band (see Supplementary Fig. S2). Immunofluorescence microscopy analysis was then performed on germinated *A. algerae* spores (Fig. 2 and see Supplementary Fig. S2 and Table S2). Full length extruded polar tubes were strongly labelled with both anti-PTP1 (Fig. 2a–c) and anti-PTP2 (Fig. 2d–f) antibodies. Interestingly, antibodies raised against PTP3 labelled a large part of the extruded polar tube except its terminal end (Fig. 2g–i). In contrast, a strong labelling restricted to the terminal end of the extruded polar tubes was observed using anti-PTP4 antibodies (Fig. 2j–l).

### Identification of a new PTP related to the PTP3 family

**Figure 3:**
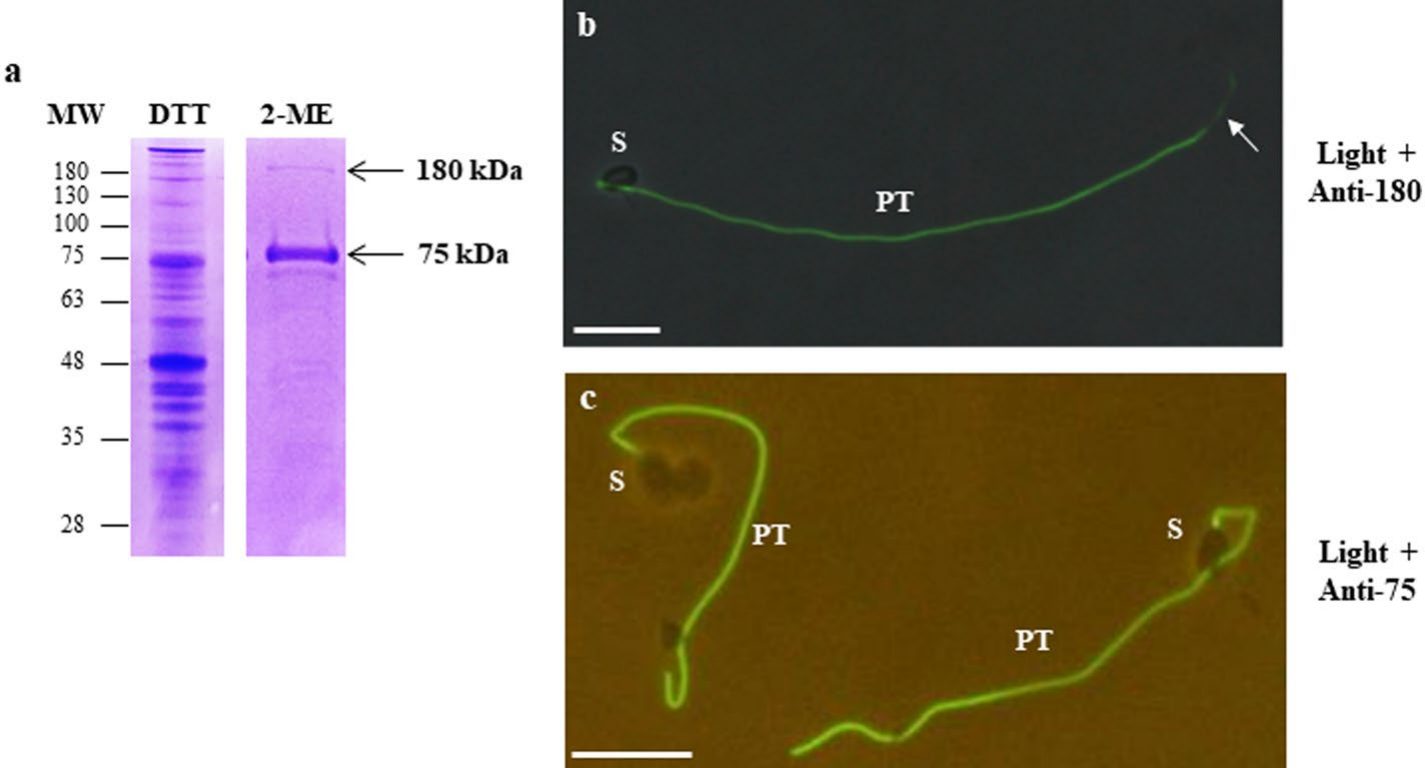
Antibodies produced against two *A. algerae* protein bands soluble in high concentration of 2-mercaptoethanol specifically labelled the extruded polar tube. (**a**) SDS-PAGE (10%) of sporal protein extracts obtained first in a buffer containing 100 mM DTT. Then, the insoluble fraction was incubated in a 50% 2-mercaptoethanol (2-ME) solution. Two protein bands running at 75 and 180 kDa (arrows) were selected in the 2-ME extract to produce mouse antibodies and were analysed in mass spectrometry (see Table S2). (**b**,**c**) IFA with mouse antisera raised against 180-kDa (**b**) and 75-kDa (**c**) protein bands. Both antisera labelled the entire polar tube of germinated *A. algerae* spores but the signal was less intensive at the end of the tube with the anti-180 kDa antiserum (arrow). Mouse antisera were used at a 1:100 dilution. Secondary antibody was Alexa 488-conjugated goat anti-mouse IgG. Merged pictures were generated with GIMP 2.10.8 software. *PT* polar tube; *S* spore; Scale bar: 10 µm.

In order to identify potential new polar tube components, differential protein extractions were performed from *A. algerae* spores using high concentrations of DTT and 2-mercaptoethanol (2-ME). The first protein extraction was done in a 100 mM DTT containing buffer. Then, the insoluble pellet was incubated in 50% 2-ME. SDS-PAGE analysis of this final fraction revealed the presence of a predominant protein band at 75 kDa (Fig. 3a). Other protein bands including one at around 180 kDa were also detected. Mouse antisera were then produced against both the 75- and the 180-kDa bands. In IFA, these antisera specifically labelled the extruded polar tube of *A. algerae* (Fig. 3b,c). Note the less intensive labelling at the terminal end of the extruded polar tube with antibodies raised against the 180 kDa protein band (Fig. 3b). As expected, mass spectrometry analysis identified both PTP1 and PTP2 in the 75-kDa protein band. Six other proteins were also found in this band, 5 of them having a predicted function (Table 2 and see Supplementary Table S3). In the 180 kDa-band, five proteins were found. Most identified peptides (43) were specific to a partial protein sequence (678 aa in length) with unknown function but related to the PTP3 family. An E3 ubiquitin ligase-like protein and another protein with unknown function were also recovered in this band. Surprisingly, no PTP3 matching peptide was identified while specific PTP1 and PTP2 peptides were found. This PTP3-like protein was thus named PTP3b. The complete CDS, obtained through RNAseq data assembly, encodes a 1197 aa protein (121 kDa, WAQ68437) with a 15 aa-predicted signal peptide (Table 1).

**Table 2:** Mass spectrometry (LC–MS/MS) analysis of the 75-kDa and 180-kDa protein bands soluble in high concentration of 2-mercaptoethanol.

Selected protein bands	Accession number or contig name	Putative function	Predicted molecular weight (kDa)	Matched peptides (number)
75 kDa	WAQ68435	PTP2	49	26*
	KI0APB13YM20FM1	Unknown	29.3	5
	KI0AEA11YJ13AHM1	Elongation factor 1α	52.9	4
	WAQ68434	PTP1	39	3
	KI0ANB25YA19FM1	Argonaute	95	3
	KI0AAA19YH14RM1	Histone H3	14.8	2
	KI0AEA15YN20FM1	20S proteasome subunit α	25.6	2
	KI0AGA12CC07FM1	Actin	41.1	2
180 kDa	WAQ68437	Putative PTP3 (PTP3b)	121	43*
	KI0AQA18YI18AHM1	E3 ubiquitin ligase-like	104.7	15
	KI0APB13YM20FM1	Unknown	29.3	8
	WAQ68435	PTP2	49	8
	WAQ68434	PTP1	39	3

*The number of matched peptides was obtained from the incomplete protein sequences before identification of the complete sequence by 5’-RACE-PCR (PTP2) or RNAseq sequencing data assembly (putative PTP3). Both nucleic and proteic sequences are listed in the Table S3. *kDa* kiloDalton.

**Figure 4:**
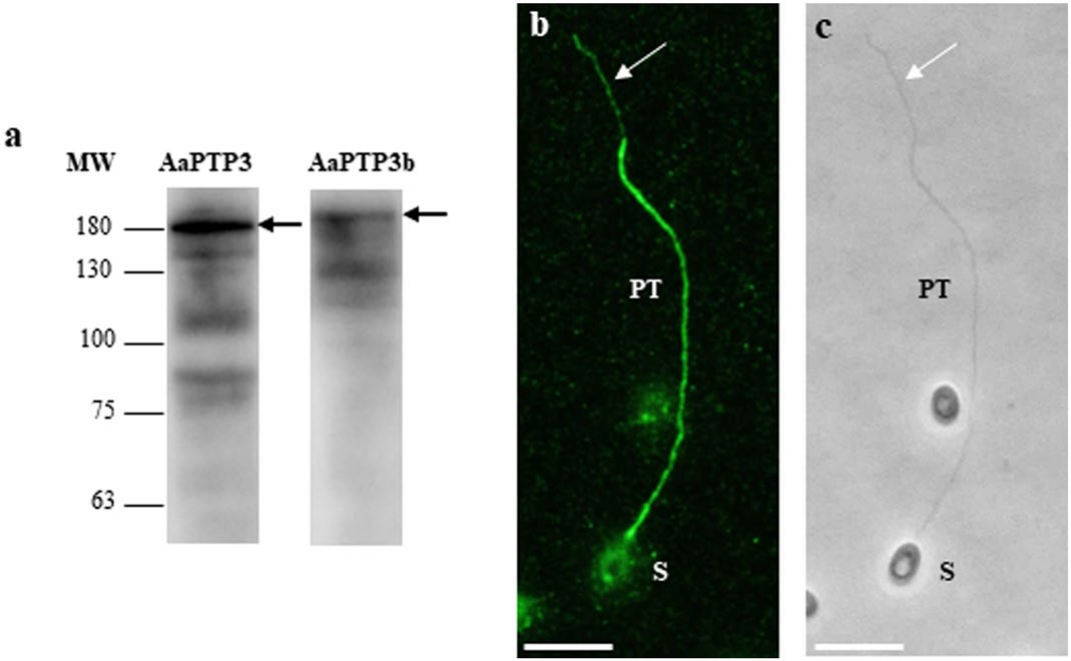
Antibodies produced against the recombinant PTP3b specifically labelled the extruded polar tube. (**a**) Immunoblotting with anti-PTP3 and anti-PTP3b against *A. algerae* total sporal protein extract (10% SDS-PAGE). PTP3b is detected at a higher molecular weight than PTP3 (arrows). The other lower detected-bands probably correspond to protein degradation. (**b**) IFA with anti-PTP3b antibodies. The entire extruded polar tube is stained but with a less intensive signal at the end-terminal part of the tube (white arrow). (**c**) phase contrast. At least 10 different fields were observed which corresponds to ~ 50 to 130 spores with their polar tube extruded. See Supplementary Fig. S2 for additional pictures illustrating the different localizations of PTPs. *PT* polar tube; *S* spore, Scale bar: 10 µm.

Sequence alignment of PTP3 and PTP3b proteins revealed that these two proteins present 29.1% of identity (see Supplementary Fig. S3). However, these PTP3 proteins present conserved biochemical characteristics including a similar high molecular weight (121 vs 127 kDa), an acidic isoelectric point close to 5 and a richness in alanine residues (Table 1). To confirm that PTP3b is a new polar tube component, antibodies were produced against a recombinant partial protein expressed in *E. coli*. As expected, these antibodies reacted with a band near 180 kDa but higher than the PTP3. In IFA, these antibodies specifically labelled the *A. algerae* extruded polar with a faint staining at the end terminal part of the tube (Fig. 4 and see Supplementary Fig. S2 and Table S2).

### MS/MS analysis of a fraction containing purified *A*. *algerae* polar tubes revealed the presence of a new PTP named PTP7

**Figure 5:**
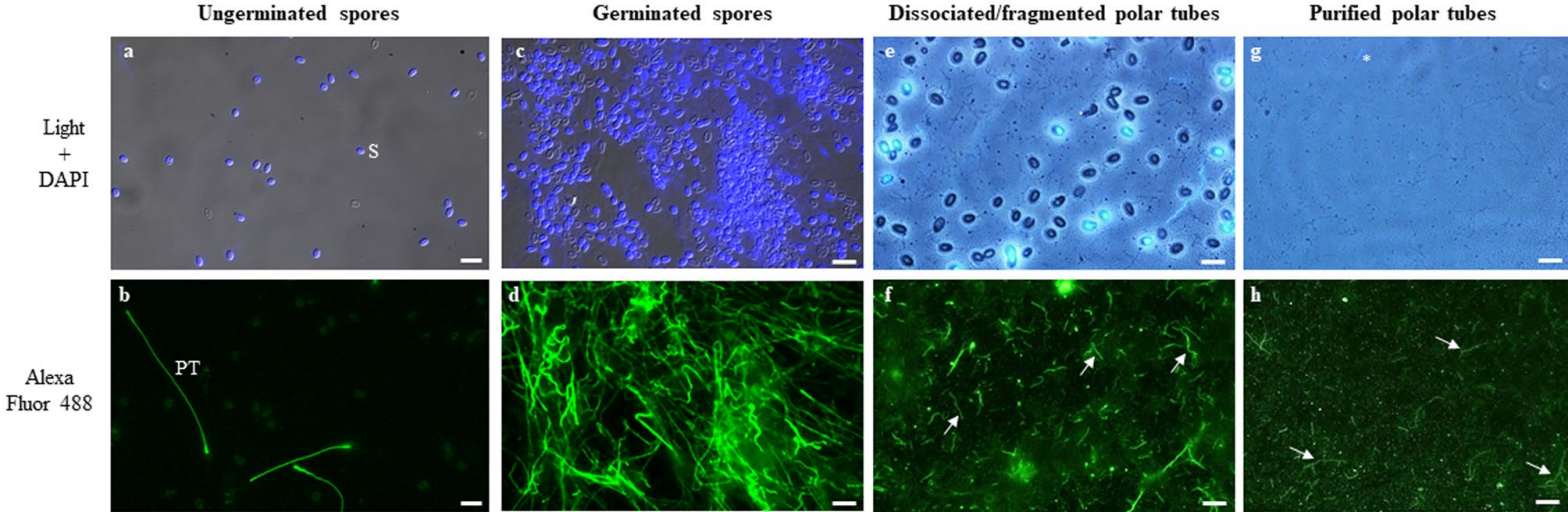
Purification of *A. algerae* polar tubes. 10^9^ spores of *A. algerae* were incubated in PBS (**a**,**b**) or in a solution containing 0.2 M NaHCO_3_-Na_2_CO_3_ at pH 9.5 for 2 h at 20 °C to stimulate the germination (**c**,**d**). Polar tubes were then dissociated from the germinated spores and fragmented (**e**,**f**). Finally, fragmented polar tubes were purified by 3 successive centrifugations (**g**,**h**). At each step, biological material was collected and analysed by DAPI staining (**a**,**c**,**e**,**g**) and by IFA (**b**,**d**,**f**,**h**) using mouse polyclonal antibodies raised against the polar tube (anti-PTP3 antibodies). Fragmented and purified polar tubes are indicated by arrows. In the final purified polar tubes fraction, some fragmented polar tubes are labelled with DAPI (**g**, *). *PT* polar tube, *S* spore. Scale bars: 10 µm.

**Figure 6:**
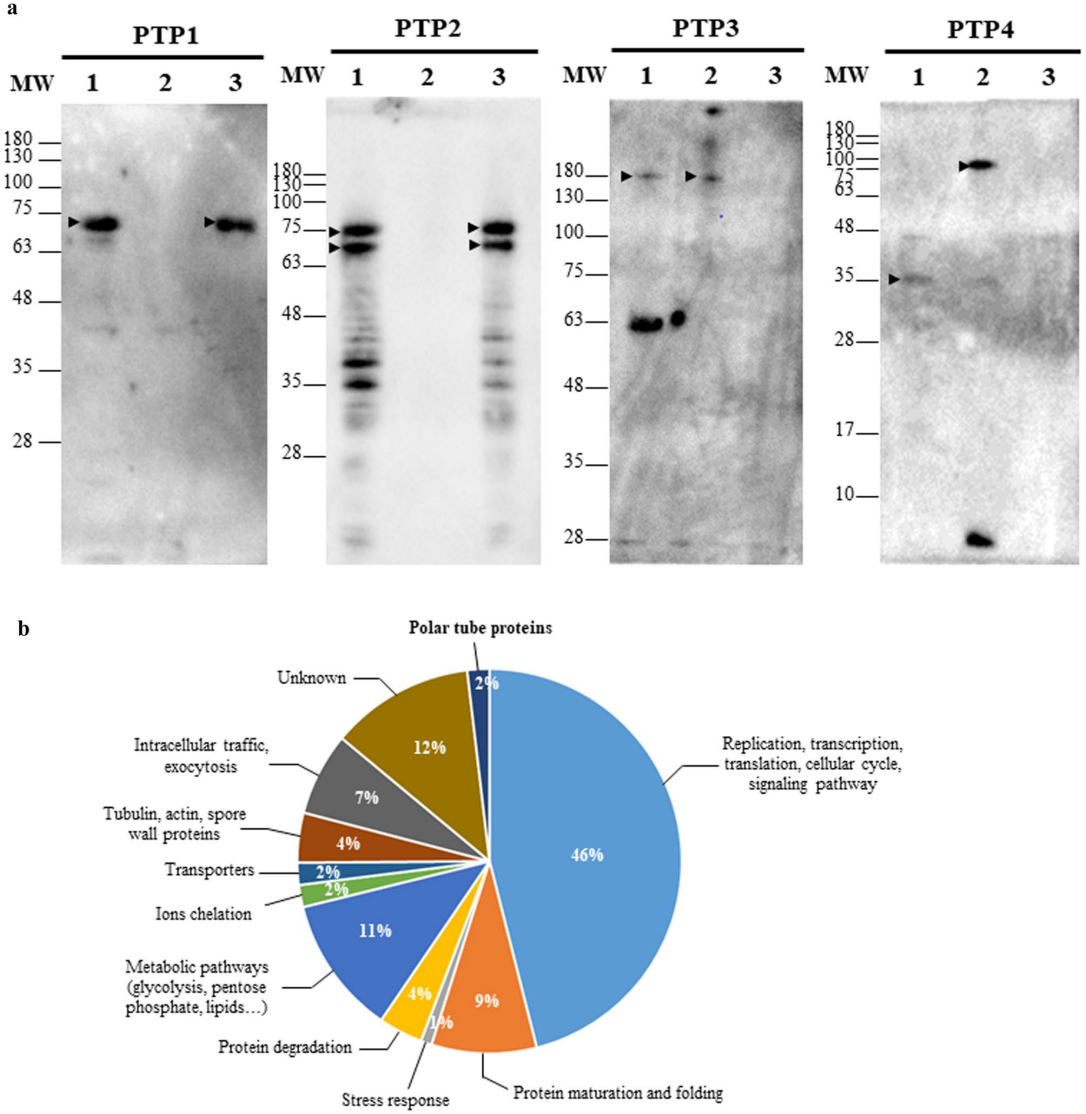
Analysis of purified polar tube fraction. (**a**) Immunodetection of PTPs in protein extracts from *A. algerae* purified polar tubes. Anti-PTP polyclonal antibodies were applied to 3 different protein extracts from purified polar tubes. The total extract (lane 1) contains proteins soluble in 2.5% SDS and 100 mM DTT. The two other extracts correspond to a differential protein extraction. Proteins from purified polar tubes were first solubilized with 2.5% SDS (lane 2), then insoluble proteins were extracted in a buffer containing 100 mM DTT (lane 3). Both PTP1 and PTP2 were only soluble in presence of reducing agents (lane 3, arrows). In contrast, anti-PTP3 and anti-PTP4 antisera detected protein bands in the SDS extract without DTT (lane 2). For PTP3, an additional band around 63 kDa was also stained in the total extract. For PTP4, the SDS extract revealed three bands of 75, 35 and < 10 kDa (lane 2). All antisera were used at a 1:200 dilution. (**b**) Functional classification and distribution of the 214 proteins identified by mass spectrometry in the purified polar tube fraction using InterProScan prediction (see Table S3).

**Figure 7:**
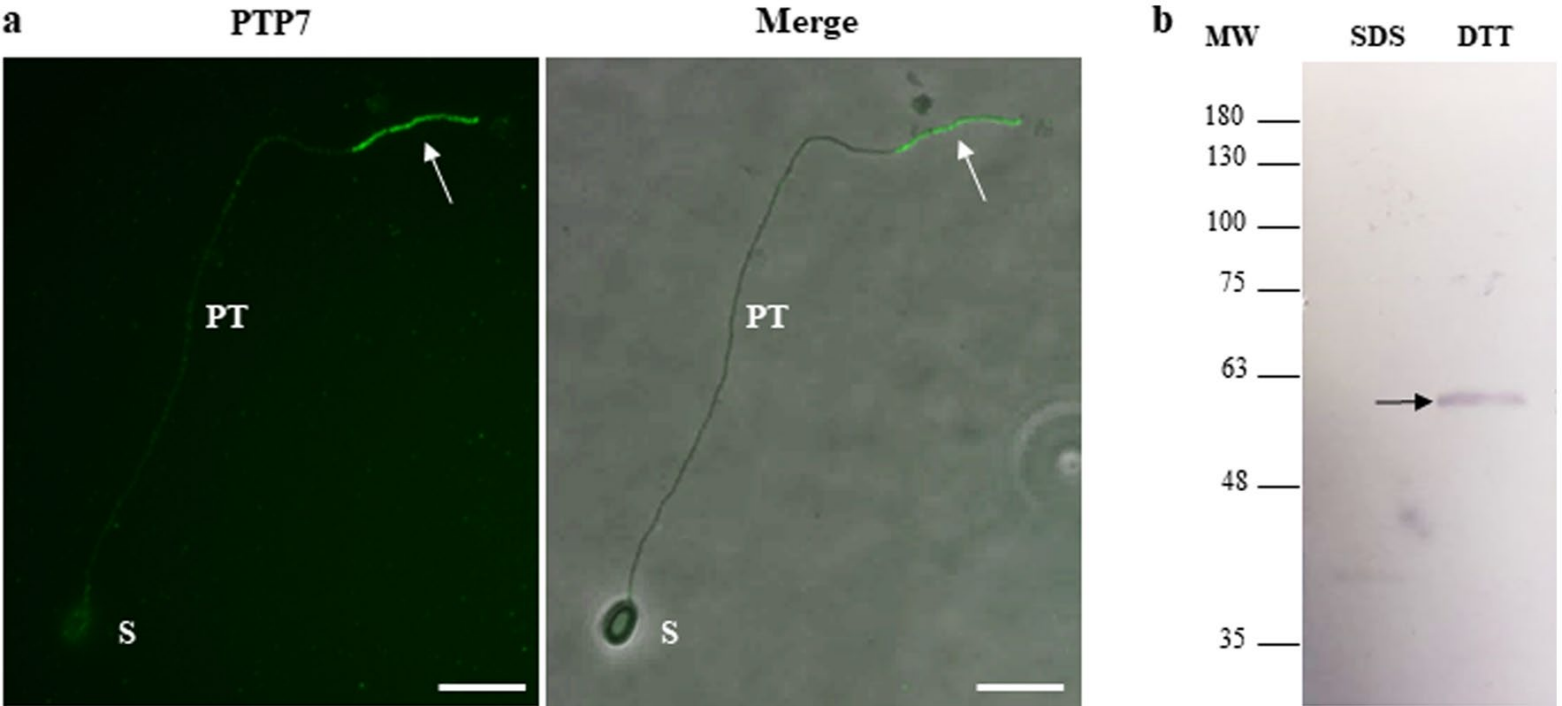
Localization of the newly identified PTP7 at the terminal end of the extruded polar tube. (**a**) IFA with anti-PTP7 mouse antiserum (dilution 1:100) revealing a specific labelling of the terminal end of the *A. algerae* extruded polar tube (arrow). Secondary antibodies were Alexa 488-conjugated goat anti-mouse IgG. At least 10 different fields were observed which corresponds to ~ 50 to 130 spores with their polar tube extruded. See Supplementary Fig. S2 for additional pictures illustrating the different localizations of PTP7. *PT* polar tube; *S* spore; Scale bar: 10 µm. (**b**) Western Blot analysis on sporal proteins solubilized first in presence of 2.5% SDS (SDS extract) and then in a 100 mM DTT- containing solution (DTT extract). The antiserum reacted with a unique *A. algerae* sporal protein band at around 60 kDa in the DTT extract (arrow).

We developed a method to dissociate and purify polar tubes from *A. algerae* germinated spores. First, *A. algerae* spores were incubated in a germination buffer at 20 °C for 2 h. The percentage of germinated spores determined by IFA using antibodies raised against the polar tube was close to 80% (Fig. 5a–d and see Supplementary Fig. S4 and Table S2). Polar tubes were then fragmented by sonication and purified by 3 successive centrifugations (Fig. 5e–h). DAPI labelling showed that this enriched PT fraction also contains sporoplasms in transit in some polar tubes. Polar tubes were then recovered in the third supernatant and proteins extracted in presence or absence of reducing agents. Western blot analysis revealed the presence of the four polar tube proteins PTP1, PTP2, PTP3 and PTP4 in the different polar tube extracts. As expected, both PTP1 and PTP2 were only detected in protein extracts with DTT whereas PTP3 and PTP4 were soluble in absence of DTT (Fig. 6a). Mass spectrometry analysis was then realized on these purified polar tube extracts. By combining the 3 extracts, a total of 214 unique proteins were identified (see Supplementary Table S3): 136 proteins in the total extract, 142 in the SDS extract and 122 in the DTT sequential extract. Although PTP1, PTP2, PTP3b and PTP4 proteins were identified in these extracts, no peptide specific to PTP3 and PTP5 was found (Table 3). Among the 214 proteins, structural proteins as spore wall proteins, ribosomal proteins, proteins involved in replication, transcription, translation, signalling and metabolic pathways were also identified (Fig. 6b and see Supplementary Table S3). More interestingly, 26 hypothetical proteins with unknown function were identified and 14 of them present a predicted signal peptide suggesting they can be secreted (Table 3). Six of these potentially secreted proteins were expressed in *E. coli* and injected in mice to produce specific antibodies. One of them, for which the complete CDS was obtained through RNAseq data assembly and corresponding to a threonine-rich protein of 417 aa (WAQ68440), was shown to localize at the terminal end of the extruded polar tube, a labelling similar to that observed with anti-PTP4 antibodies (Fig. 7a). In Western blot, these antibodies detected a single protein band around 60 kDa only in protein extracts containing reducing agents (Fig. 7b). This new polar tube component named PTP7 had no homology with other PTPs and proteins from databases. Only one orthologous protein was found in the closely related microsporidian species *Tubulinosema ratisbonensis*.

**Table 3:** PTPs and proteins with unknown function identified by mass spectrometry analysis in purified polar tube protein extracts.

Protein or contig name with gene coordinates	Number of amino acids	Predicted size (kDa)	Number of corresponding peptides	Signal peptide	Orthologs in other microsporidia
PTP1	407	39	2	+	Yes
PTP2	535	49	19*	+	Yes
PTP4	254	28	3	+	Yes
PTP3b	1197	121	20*	+	Yes
KI0ANB26YD05AHM1:c(2211-4190)	659	72.3	30	+	Yes
KI0AFA5DG12FM1:c(2847-3884)	345	39.7	28	–	No
KI0ALA15YI13FM1:c(2240-3841)	533	61.9	16	+	No
KI0AAD35CC01FM1:621-1507*	nd	nd	12	–	No
**KI0ABA57YA11FM1:c(642-2246)**	**534**	**60.8**	**11**	** + **	**No**
KI0AGA7DE03FM1:320–1243*	nd	nd	8	+	Yes
KI0ANB24YI16AHM1:c(1635-3116)	493	55.4	8	+	Yes
KI0AEA10YM04AHM1:c(3308-4249)	313	36.9	8	–	No
KI0AAD45CE04FM1:2-472*	**nd**	**nd**	**7**	**nd**	**No**
KI0AGA16AB12AHM1:2031-2963	310	33.9	6	+	Yes
KI0ALA3YH04FM1:1160-3235	691	79.3	6	+	No
*PTP7*	*417*	*45.5*	6*	* + *	*Yes*
KI0AEA7YF20FM1:c(2933-3760)	275	31.2	5	–	No
**KI0APB13YM20FM1:c(434-1237)**	**267**	**27.6**	**5**	** + **	**No**
KI0AIA13YP22AHM1:c(7015-7737)*	nd	nd	5	nd	Yes
KI0AEA19YB16FM1:c(1-533)*	**nd**	**nd**	**5**	** + **	Yes
**KI0ANB9YM14AHM1:c(2144-3451)**	**435**	**46.9**	**5**	** + **	**No**
KI0AAD70DA10FM1:c(1-640)*	nd	nd	4	nd	Yes
KI0AGA15DF12FM1:1-1002*	nd	nd	3	nd	Yes
KI0AAD12AH02AHM2:1916-2944*	nd	nd	2	–	No
KI0ALA20YE02FM1:7644-9241*	nd	nd	2	–	Yes
KI0ANB24YE20FM1:c(282-1136)	284	33	2	–	Yes
KI0ALA2YJ16AHM1:c(1-1073)*	nd	nd	2	+	No
KI0AKA10YI05AHM1:1591-2230*	nd	nd	2	–	Yes
KI0AIA10YM10FM1:54-797	247	26	2	+	No
KI0AAA20YM17RM1:382-948	188	20	2	+	Yes

In bold, proteins with unknown function selected to produce antibodies. The newly identified PTP7 is indicated in italic, the identified sporoplasm protein is underlined (KI0AAD45CE04FM1:2-472).

*Sequence incomplete in C-terminal or in N-terminal. The number of amino acids is indicated for complete proteins and the predicted size for mature proteins. The number of corresponding peptides is the higher number identified in at least one extract. ( +) Presence of a predicted signal peptide. (–) No predicted signal peptide. Identification of potential orthologs was performed by BLASTp search against NCBI and MicrosporidiaDB databases. (nd) not determined because of partial sequence.

## Discussion

The polar tube is a highly specialized organelle involved in the unique host cell invasion process used by microsporidian parasites. Upon appropriate environmental stimulation, this hollow tubular structure which is coiled around the interior of the spore is suddenly extruded and used to deliver the infectious sporoplasm into the host cell cytoplasm^28,29^. Five polar tube proteins named PTP1 to PTP5 were identified and shown to be present in a majority of microsporidian species but little is known regarding polar tube formation and the function of the components that make up this structure^15,26^. Although PTPs share common characteristics within each family (e.g. proline richness for PTP1, basic pI for PTP2, solubility properties, conserved gene order for *ptp1* and *ptp2*), they are characterized by a high level of interspecies amino acid sequence variability. Thus, identification of PTPs often requires a combination of sequence similarity searches in databases and comparison of both biochemical properties and gene organization. For example, the syntenic relationship of *ptp1* and *ptp2* genes facilitated their identification despite their strong sequence divergences in the insect microsporidia *Antonospora locustae* and *Paranosema grylli*^22^. In the present study, we succeeded in the identification of the genes coding for orthologous proteins belonging to the 5 PTP families in *Anncaliia algerae*. As expected, these proteins in particular the major PT component PTP1 are characterized by a high degree of amino acid sequence divergence when compared to their orthologs in other microsporidian species. Although *ptp1* and *ptp2* genes were found at the same genomic locus in numerous microsporidian genomes including those of *Encephalitozoon* spp.^21^, *A. locustae* and *P. grylli*^30^, this tandem organization does not seem to be conserved in *A. algerae.* Surprisingly, analysis of *A. algerae* genomic data also revealed the presence of three partial sequences coding for PTP2 but only the full sequence of one of them was obtained by RACE PCR (see Supplementary Fig. S1). These PTP2-related proteins are characterized by a highly conserved C-terminal region with many cysteine residues and contain a divergent N-terminal extension glycine- and serine-rich, as previously observed in *A. locustae* and *P. grylli*^30^. The presence of multiple PTP2-like proteins was confirmed by western blot analysis as anti-PTP2 antibodies reacted with several protein bands (Fig. 1). A recent study performed in the silkworm parasite *Nosema bombycis* has revealed the presence of a new PTP named NbPTP6^16^. This PTP rich in histidine and serine residues was demonstrated to bind to the host cell surface but seems to be less conserved in Microsporidia. Besides we were unable to find an orthologous protein in *A. algerae*.

To complete our knowledge on the composition of the polar tube in *A. algerae*, we thus combined two approaches. The first one was based on the solubility properties of the polar tube and the second approach involved the purification and proteomic analysis of an extruded polar tube fraction. As the polar tube has been demonstrated to be fully dissociated in presence of reducing agents^15^, differential protein extractions were done using high concentrations of DTT and/or 2-ME. Two major bands migrating at 75 and 180 kDa were selected from a 50% 2-ME protein extract. As expected, the 75-kDa protein band contains both PTP1 and PTP2, these two proteins having been demonstrated to co-migrate in SDS-PAGE (Fig. 1). More surprisingly, PTP3 was not identified in the 180-kDa protein band. However, proteomic analysis of this band revealed the presence of a PTP3-like protein, named PTP3b. PTP3 and PTP3b are highly divergent (only 29% of sequence identity, see Supplementary Fig. S3) but they share common characteristics including a high molecular weight, an acidic pI and a glutamate and alanine richness. Two PTP3 encoding genes were also identified in the recently sequenced genome of *T. ratisbonensis*, a species closely related to *A. algerae*^31^ or in the mosquito parasite *Edhazardia aedis*, whereas a single *ptp3* gene was identified in all the other microsporidian genomes sequenced so far^15^.

The second approach to identify new *A. algerae* PTPs consisted in developing a method to purify polar tubes from germinated spores. A similar strategy was recently used in *Nosema bombycis* and ten unknown secreted proteins without any conserved domain were identified as potential novel PTPs but the PT localization of these proteins was not demonstrated^32^. In our study, we first selected and optimized a germination protocol for *A. algerae*^26^ leading to ~ 80% of spores with extruded polar tube. Polar tubes were then dissociated from the germinated spores, fragmented by sonication and purified by successive centrifugations. PTP1, PTP2, PTP3, PTP3b and PTP4 but not PTP5 were identified in the polar tube extracts as demonstrated by western blot (Fig. 6) and/or mass spectrometry (Table 3) analysis. Among the 214 proteins identified by mass spectrometry, we also found 14 secreted proteins with unknown function that could represent new potential PTPs, but mouse antibodies were only produced against 6 selected proteins. IFA analyses revealed that one of these proteins corresponds to a new sporoplasm protein (not shown) while another is a new PTP, named PTP7 presenting no homology with the other PTP families, including the recently identified PTP6 in *N. bombycis*. Homology search only identified an orthologous PTP7 in *T. ratisbonensis*, suggesting that this PTP7 is absent in most microsporidian genomes or is highly divergent which makes its identification difficult. We can thus hypothesize that certain PTPs could be specific to some microsporidian species probably in connection with their host specificity. Analysis of purified PT extracts also revealed the presence of several spore wall proteins (SWPs). This can be explained by the fact that interactions between PTPs and SWPs and the role played by some SWPs in the polar tube formation and orientation have been previously described in *N. bombycis*^33,34^. For example, SWP9 was shown to be a scaffolding protein that anchors and holds the polar tube but also tethers the polar tube to the spore wall^35^. Thus, polar tube orientation, arrangement and anchorage to anchoring disk would be dependent on PTP-SWP interactions. More surprisingly, numerous proteins involved in replication and translation processes, cellular signalling or metabolism were also found in the purified polar tube fraction. They probably correspond to proteins from the sporoplasm in transit in extruded polar tubes (as demonstrated by DAPI labelling), which thus contaminated polar tube fractions. Similarly, a large number of proteins, including this kind of proteins, were also identified from *N. bombycis* purified polar tubes^32^. To support our hypothesis, we could analyse the protein content of purified *A. algerae* sporoplasms using the protocol recently developed for the purification of *N. bombycis* sporoplasms^36^.

Production of antibodies against *A. algerae* PTPs allowed us to analyse their solubility and to precise their localization. PTP1, PTP2 and PTP7 were only recovered in protein extracts containing DTT, as previously described for all identified PTP1 and PTP2^21,22,30^. This is coherent with the presence and the conservation of several cysteine residues most likely involved in intra- and/or intermolecular disulfide bonds required for polar tube assembly^24^. Unlike PTP1 and PTP2, PTP3, PTP3b and PTP4 were soluble in SDS alone as described for *E. cuniculi* PTP3^23^. Proteins from the PTP3 family contain a few numbers of cysteine residues and have been suggested to interact with other PTPs via ionic bonds during the formation of the PT^23,24^. PTP4, with a predicted size of 28 kDa, is detected at both 35 and 75 kDa in absence of reducing agent but only bound to the 35-kDa band in the DTT-soluble extract. We hypothesized that the highest band detected without DTT could correspond to homodimers established via disulfide bonds. However, this hypothesis needs to be tested by yeast two hybrids or immunoprecipitation experiments. Finally, western blot analysis revealed that PTP1, PTP2 and the two PTP3 proteins displayed molecular weights larger than their predicted sizes. Indeed, both PTP1 (39 kDa) and PTP2 (49 kDa) co-migrate around 75 kDa, while PTP3 (127 kDa) and PTP3b (121 kDa) are detected in higher but different molecular weight bands close to 180 kDa. A migration defect was also observed for the newly identified PTP7 with a single band migrating at ~ 55/60 kDa for a predicted size of 45 kDa. For PTP1, the very high content in proline residues (32%) could contribute to this anomalous migration, also observed for most identified PTP1^18,21,22^, as it is well known that proline-rich proteins run slower during SDS-PAGE. More likely, the cause of aberrant migration could be due to post-translational modifications such as glycosylation. Indeed, all PTP1-3 and PTP7 contain numerous potential O-glycosylation sites and some proteins of the PTP1 family were shown to be O-mannosylated^20,22^. It has been suggested that such modifications would be involved in the adherence of the PT with the host cell surface through interactions with mannose-binding receptors^19^. However, the glycosylation status of the other PTPs needs to be clarified.

Our study also revealed that the 6 PTPs of *A. algerae* showed different localizations in the extruded polar tube. As described in other species, PTP1 and PTP2 proteins are located uniformly along the entire extruded polar tube. This is consistent with the fact that PTP1 is considered as the major component of the polar tube and was shown to interact with itself and with both PTP2 and PTP3 in the formation of the polar tube in *E. cuniculi*^24^. In contrast, PTP3 is present on a wide part of the fully extruded polar tube except at its terminal end. However, when the sporoplasm is still in the spore or in transit in the extruded PT, the tube was totally labelled by anti-PTP3 antibodies in agreement with (i) polar tube ‘everting like the finger of a glove’ model^37^ and the fact that infectious material initiates its transfer while only 50% of the tube is extruded^14^ (see Supplementary Fig. S2). Thus, when the sporoplasm transfer starts, the PT extremity would not be accessible leading to a full-length polar tube staining with anti-PTP3 antibodies. In contrast to PTP3, PTP3b is present on the entire polar tube with a less intensive labelling at its extremity as observed with PTP3 from *Nosema pernyi*^38^. Finally, antibodies to PTP4 and the newly identified PTP7 demonstrate a similar localization of both proteins restricted to the tip of the polar tube, corresponding to the region that was not labelled with anti-PTP3 antibodies. However, some extruded polar tubes were not labelled with these antibodies, the absence of labelling being also observed for germinated spores with their sporoplasm still inside the spore or in transit in the extruded polar tube. In these cases, the polar tube was not fully extruded and its extremity would not be accessible to the antibodies (see Supplementary Fig. S2 and Table S2). Our IFA data need to be completed by TEM immunolabeling, in particular to precise the location of the different PTPs when the polar tube is still coiled within the spore. In *E. hellem*, a monoclonal antibody to PTP4 labelled only the tip of polar tube, and it was demonstrated that an epitope of this protein can interact with the host cell transferrin receptor 1 to initiate the invasion process^27^. We can hypothesize that PTP4 could play a similar role in *A. algerae*. The same localization of PTP7 also argues for a role of this PTP in the recognition of potential host cell receptor during the process of invasion. To conclude, our study provides new advances in polar tube composition, but the interactions between PTPs required for polar tube assembly and their respective role during the invasion process remain enigmatic.

## Materials and methods

### Cell culture and *A*. *algerae* spore production

The sequenced *A. algerae* isolate used in this study^39^ was kindly provided by Pr W. A. Maier (University of Sigmund-Freud, Bonn) and corresponds to the microsporidium isolated by Dr A. Undeen from its original mosquito host (*A. stephensi*). This isolate was propagated in vitro in Rabbit Kidney (RK13, ATCC CCL-37) or Human Foreskin Fibroblast cells (HFF, ATCC SRC-1041), in minimum essential medium (MEM) supplemented with 5% fetal calf serum, 2 mM l-glutamine, 0.25 µg.mL^−1^ amphotericin B and 25 µg.mL^−1^ gentamicin, at 30 °C in a 5% CO_2_ atmosphere. Spore-containing supernatants were collected every 72 h. After centrifugation for 3 min at 6000×*g*, spores were washed 3 times in PBS buffer and stored at 4 °C.

### Identification of polar tube protein encoding genes in the *A*. *algerae* genome

PTPs sequences described in other microsporidian species and available in NCBI and/or MicrosporidiaDB databases (https://microsporidiadb.org/micro/app) were used as a query. Using these sequences, a search by sequence homology was realized against the genome of *A. algerae* using BLAST with default settings. Sequences identified in the *A. algerae* genome were then characterized with different bioinformatic tools. N-terminal signal peptides were predicted using a combination of SignalP-5.0 (http://www.cbs.dtu.dk/services/SignalP/), TargetP-2.0 (http://www.cbs.dtu.dk/services/TargetP/) and Phobius (https://phobius.sbc.su.se/) tools. Theoretical isoelectric point, molecular weight and amino acid composition were determined with ExPASy ProtParam tool (https://web.expasy.org/protparam/). Potential O-glycosylation sites were predicted with NetOGlyc 4.0 server (http://www.cbs.dtu.dk/services/NetOGlyc/). Functional domains and gene ontology terms were determined with InterPro 86.0 tool (https://www.ebi.ac.uk/interpro/). Multiple sequence alignments were performed using the ClustalOmega software (https://www.ebi.ac.uk/Tools/msa/clustalo/).

### Production of recombinant *A*. *algerae* PTPs in *Escherichia coli*

*A. algerae* DNA was released from 1.7.10^8^ spores using the NucleoSpin® Soil kit (Macherey–Nagel). Sequences encoding PTPs of interest were amplified by PCR with 130 ng of DNA, 500 nM of each specific primer (see Supplementary Table S1), 0.2 mM dNTPs and 1.25 U of GoTaq® DNA polymerase (Promega). PCR amplifications were performed using a ProFlex PCR System thermal cycler (Applied Biosystems). After denaturation at 94 °C for 2 min, 30 cycles were run with 30 s of denaturation at 94 °C, 30 s of annealing between 56 and 60 °C and 40 to 90 s of extension at 72 °C. PCR products were separated on 1% agarose gel and purified using the PCR clean-up gel extraction kit (Macherey–Nagel). Then, PCR products were digested with corresponding restriction enzymes (see Supplementary Table S1, Promega) and cloned into the expression vector pGEX-4T1 in frame with glutathione-S-transferase (GST) and a 6 histidine tag at the C-terminal. The resulting recombinant plasmids were introduced into the *E. coli* BL21^+^ strain. After induction at 37 °C with 1 mM IPTG for 4 h, bacterial proteins were solubilized in 2.5% SDS and 10% 2-mercaptoethanol then analysed by SDS-PAGE. Each recombinant protein was then purified on glutathione beads according supplier’s instruction (Glutathione Sepharose® 4B, GE Healthcare).

### Identification of a full length PTP2 encoding gene by 5’-RACE-PCR

HFF cells were infected with 10^7^ spores of *A. algerae* and total RNAs were extracted at 72 h post-infection using Trizol (Ambion) and then purified using the Qiagen RNeasy Mini Kit (Qiagen). The 5′cDNA end of *ptp2* was characterized using SMARTer® RACE 5’/3’ kit according to the manufacturer’s recommendations (Takara Bio). The reverse transcription (RT) reaction step was performed with 1 ng of total RNAs from infected cells using *ptp2c* specific primer (5’-GTATGGATCCAATGTGGTTG-3’) according to the manufacturer’s recommendations. 5’-RACE-PCR was realized with the thermal cycler ProFlex PCR System with following parameters: 10 cycles with a denaturing step of 30 s at 94 °C, 30 s of annealing at 68 °C and an elongation step of 1.5 min at 72 °C followed by 30 cycles with a denaturing step of 30 s at 94 °C, 30 s of annealing at 64 °C and an elongation step of 1.5 min at 72 °C followed by a final elongation of 10 min at 72 °C. 5’-RACE-PCR products were analysed on 1% agarose gel, purified with the QIAquick Gel Extraction kit (Qiagen), cloned in the pGEM®-T Easy (Promega) and sequenced (Eurofins Genomics).

### Antibody production

Polyclonal antibodies against *E. coli* expressed recombinant proteins (PTP2, PTP3, PTP3b, PTP4, PTP5 and PTP7) were produced in CD1 mice from purified proteins bands separated by SDS–PAGE (see below). Polyclonal antisera were also produced against *A. algerae* 75- and 180-kDa bands isolated from differential protein extractions. Following Coomassie blue staining, protein bands were excised and crushed in an elution buffer (50 mM Tris–HCl, 150 mM NaCl, 0.1 mM EDTA pH 7.5). Mice were injected intraperitoneally with sample homogenized (vol/vol) with Freund’s complete adjuvant (Sigma) for the first injection. Additional injections were realized with sample homogenized with Freund’s incomplete adjuvant (vol/vol, Sigma) at day 14, 21, 28 and 35. Sera were collected 1 week after the last injection and stored at − 20 °C. Antibodies against PTP1 were produced in rabbits against two synthetic peptides located in the N-terminal part (CDEGGSPGSGKPSTLV, aa30-aa45) and the C-terminal part (CEAPQNEGKTEEAPP, aa343-aa357) of the protein (Eurogentec).

### Purification of *A*. *algerae* polar tubes

10^9^ spores of *A. algerae* were resuspended in 1 mL of a germination solution (0.2 M NaHCO_3_-Na_2_CO_3_ pH 9.5) and incubated for 2 h at 20 °C. Then, germinated spores were washed twice with 1 mL of PBS and centrifuged at 10,000×*g* for 2 min. The pellet was resuspended in PBS and polar tubes were fragmented by sonication during 40 s at 60 Hertz. Next, 3 successive centrifugations (2000×g 2 min) allowed to separate fragmented polar tubes (supernatant) and spores (pellet). The third supernatant was collected, centrifuged for 10 min at 16,000×*g*, washed with 1 mL of PBS and centrifuged again at 16,000×*g* for 10 min to pellet fragmented polar tubes. The purity of each fraction was estimated by IFA using antibodies directed against the polar tube (anti-PTP3), as described in the IFA section. Presence of sporoplasms in the purified polar tubes was checked using DAPI staining as described in the IFA section. In each condition 10 fields were analysed and both germinated spores and total spores were counted. The percentage of germinated spores was then calculated (see Supplementary Fig. S4 and Table S2). Proteins were extracted as described in the next section and analysed by SDS-PAGE and by western blot using anti-PTP antibodies but also by mass spectrometry.

### *A*.* algerae* protein extractions and western blotting

Total protein extracts from 10^9^ *A. algerae* spores or purified polar tube fractions were solubilized using a buffer containing 2.5% SDS and 100 mM DTT. Spores or polar tubes were thus disrupted in this buffer by 10 freezing–thawing cycles in liquid nitrogen followed by boiling for 15 min. After centrifugation (14,000×*g* for 2 min), total soluble proteins (ET) were collected in the supernatant. Differential protein extractions were also done by treating spores or purified polar tubes with 2.5% SDS (SDS extract) and then with a lysis buffer containing 2.5% SDS and 100 mM DTT (DTT extract) or with 2.5% SDS and 100 mM DTT followed by a lysis buffer containing 2.5% SDS-50% 2-mercaptoethanol (2-ME). *A. algerae* protein samples were analysed by SDS-PAGE on 8%, 10% or 12% polyacrylamide gels depending on the molecular weight of proteins and stained by Coomassie blue. For immunoblotting studies, proteins were transferred onto polyvinylidene difluoride (PVDF) membranes (Merck Millipore). After blocking for 1 h in 5% milk in PBS, membranes were incubated for 3 h with mouse or rabbit antisera diluted at 1:200 in PBS-0.1% Triton X-100 followed by 3 washes in PBS-0.1% Triton X-100. Membranes were then incubated with a 1:2500 dilution of alkaline phosphatase-conjugated or peroxidase-conjugated goat anti-mouse or anti-rabbit Ig G (H + L) (Promega) for 1 h at room temperature and revealed respectively with NBT/BCIP substrates or ECL mix and with ChemiDoc system (BioRad).

### Indirect immunofluorescence assay (IFA) on germinated spores

Antibodies raised against both recombinant PTPs or *A. algerae* proteins were tested by indirect immunofluorescence assay on germinated spores. Spore germination was stimulated on 10^7^ spores of *A. algerae* using 0.2 M NaHCO_3_-Na_2_CO_3_ pH 9.5 solution. After incubation at 20 °C for 2 h, 50 µL of germinated spores were put on glass coverslips, dried overnight and fixed with methanol at -20 °C for 20 min. Following fixation, slides were saturated with PBS-1% BSA and then incubated at room temperature for 3 h with antibodies diluted at 1:100 in PBS-0.1% Triton X-100. After 3 washes in PBS-0.1% Triton X-100 during 5 min, slides were incubated with secondary antibody diluted at 1:1000 in PBS-0.1% Triton X-100 during 1 h (anti-mouse or anti-rabbit Alexa Fluor 488, Invitrogen). After 3 washes in PBS-0.1% Triton X-100 and a wash in PBS, smears were incubated for 5 min with DAPI (0.1 mg.L^−1^). Finally, slides were mounted on glass slide with ProLong™ Diamond Antifade Mountant (Molecular Probes). Slides were observed under a Leica DM IRB epifluorescence microscope and images were superimposed with GIMP 2.10.8 software. At least 10 different fields were observed which corresponds to ~ 50 to 130 spores with their polar tube extruded.

### Protein identification by Nano-LC–MS/MS and analysis

The preparation of protein bands was carried out according to Zhu et al.^40^. The peptides extract adjusted exactly to 30 µL with a recovery solution (H_2_O/ACN/TFA-94.95/5/0.05) was analysed by nano-LC-MS/MS using an Ultimate 3000 system coupled to a QExactive HF-X mass spectrometer (MS, Thermo Fisher Scientific). One μL of hydrolyzate was first preconcentrated and desalted during 6 min on a C18 pre-column (equilibration Trifluoroacetic Acid 0.05% in H_2_O—flow rate 30 µl/min) then analysed on a C18 Acclaim PepMap 100 column (equilibration 96% solvent A (99.9% H_2_O, 0.1% formic acid)—flow rate 300 nl/min) with a gradient of solvent B (99.9% acetonitrile, 0.1% formic acid) of 5 to 32% in 50 min. Eluted peptides were electrosprayed through a nanoelectrospray ion source in the mass spectrometer operating in top N data dependent mode (DDA). Raw data processing was carried out with the Proteome Discoverer Sofware v1.4 and MS/MS ion search was performed with Mascot v2.5.1 (http://www.matrixscience.com) against the local databank Local_annc_alg database^39^ with the following parameters: precursor mass tolerance of 10 ppm, fragment mass tolerance of 0.05 Da, maximum of two missed cleavage sites of trypsin, carbamidomethylation (C), oxidation (M) and deamidation (NQ) set as variable modifications. Protein identification was validated when at least two peptides originating from one protein showed statistically significant identity above Mascot scores > 21 with a False Discovery Rate of 2.4% (default significance threshold p < 0.05). Ions score is − 10 log(P), where P is the probability that the observed match is a random event. From the protein list identified, functional domains and gene ontology terms were determined with InterPro 86.0 tool (https://www.ebi.ac.uk/interpro/).

### RNAseq sequencing and ptp genes assembly

Total RNAs were extracted from HFF cells infected with *A. algerae* (72 h post-infection). mRNAs were retrotranscribed with the Illumina TruSeq stranded mRNA kit (Illumina) then bound to adapters and fixed on the flowcell plate before PCR amplification. A paired end Illumina sequencing (2 × 50 bp) with the NovaSeq6000 sequencer (Illumina) was done (Fasteris). A total of 1.7 × 10^9^ reads was obtained. The quality of reads was controlled with fastQC software (Galaxy version 0.72) and reads were then mapped against the human genome (version HG38 NCBI 18.05.21) with HISAT2 software (Galaxy version 2.1.0) to remove mRNAs corresponding to host cells. Two de novo assemblies of the 252 × 10^6^ remaining reads were done with Megahit (Galaxy version 1.1.3.5; default settings; minimum multiplicity set to 2 for filtering) and Trinity (Galaxy version 2.8.4; default settings (k value = 25) and a minimum of 2 K-mers). A total of 2793 and 4833 contigs were obtained with these two softwares, respectively. Using these two sequences set as databases, genes coding for PTPs were identified by a local BLASTn approach with a word size of 11 nucleotides using our previously identified *A. algerae ptp* sequences as queries.

### Ethical approval

The study was conducted in accordance with the relevant ARRIVE guidelines and regulations. Mouse experiments were conducted according to French and European directives for the use and care of animals for research purposes and were approved by the “Comité d’Éthique pour l’Expérimentation Animale en Auvergne” (project agreement #2015120111273779).

## Supplementary Information

Supplementary Figure S1.

Supplementary Figure S2.

Supplementary Figure S3.

Supplementary Figure S4.

Supplementary Table S1.

Supplementary Table S2.

Supplementary Table S3.

## Data Availability

The assembled contig sequences for *A. algerae* are available in the NCBI Assembly database (www.ncbi.nlm.nih.gov/assembly/) under the accession number GCA_000313815.1. Accession numbers for complete *A. algerae* PTPs are WAQ68434 (PTP1), WAQ68435 (PTP2), WAQ68436 (PTP3), WAQ68437 (PTP3b), WAQ68438 (PTP4), WAQ68439 (PTP5) and WAQ68440 (PTP7). RNA-seq raw data from *A. algerae*-infected HFF cells have been submitted to NCBI with accession code SAMN32904311. Full amino acid sequences of proteins identified by mass spectrometry are available in the Supplementary Table S3. The mass spectrometry proteomics data have been deposited to the ProteomeXchange Consortium via the PRIDE^41^ partner repository with the dataset identifier PXD040773.
