# Supplementary material for: Identification and localization of polar tube proteins in the extruded polar tube of the microsporidian Anncaliia algerae
Source: Sci Rep. 2023 May 30;13:8773. doi: 10.1038/s41598-023-35511-y (PMC10229552; doi:10.1038/s41598-023-35511-y)
Supplement: Supplementary file 1 — Supplementary Figure S1. [file 41598_2023_35511_MOESM1_ESM.pdf]

Figure S1. Alignment of PTP2 amino acid sequences identified in *A. algerae*. Corresponding proteins were incomplete in their N-terminal region. A 5'-RACE-PCR approach allowed to identify the lacking sequence coding for the N-terminal part of PTP2c (sequence in red). PTP2c predicted signal peptide is boxed. PTP2 proteins have a conserved C-terminal region and a variable N-terminal region due to insertions/deletions events in repeated motifs rich in glycine and serine residues. Identical amino acids are highlighted in grey and the eight cysteine residues in conserved position are highlighted in black. The two sequences underlined in black correspond to regions that were selected for the expression of recombinant polypeptides in *E. coli*. Specific peptides for each PTP2 were identified by mass spectrometry analysis and are underlined in red. This confirms the presence of the three PTP2 in the *A. algerae* polar tube. Amino acids are numbered on the right. The accession number for the full-length PTP2c is WAQ68435.1. The contig names for PTP2a and PTP2b are KI0AQA2YO04FM1_3..1481 and KI0ALA25YH17FM1:c(216..1571), respectively and are available under the accession number GCA_000313815.1.

```
PTP2c      MRKPVLLAILVFVYQSQAGYSWSSSSRSSSGGSRGGGSSGGASSGGGSGGASMSGVSSGG   60
PTP2b      ------------------------------------------------------------    0
PTP2a      ------------------------------------------------------------    0

PTP2c      SSMSFSGGSSGGASSGGSSSHGSGGSGSGSSGHGSGSGGGAPGKSSGSESGSGAPGGGS  120
PTP2b      ---------------------GGSGSGSGSGSGHGSGSGGGAPGG----GSGSGAPGGGS   35
PTP2a      --------------------------GSGSGSGHGSGSGGGAPGGGSGHGSGSGTPGGGS   34

PTP2c      GSGSGSGSGHGSGSGGGAPGGGSGNGSGSGSGHGSGSGGGAPGGGSGSGAPGGGSGGGSG  180
PTP2b      GGGSGSGSGHGSGSGGGAPGGGSGNGSGAGSGNG-----------SGSGAPGGGSGSGPG   84
PTP2a      GSGPGSGSGHGSGSGGGAPGGGSDSGSGAGSGAQGGSSAGE-------------------   75

PTP2c      SGSGHGSGSGGGAPGGGSDSGSGAGSGAQGGSSAGEGSGAKGSGSAGEGSGAQGGSSAG-  239
PTP2b      SGSGHGSGSGGGAPGGGSDSGSGAGSGAQGGSSAGEGSGAKGSGSAGEGSGAQGGSSAGE  144
PTP2a      ---------GSGAKG---SGSAGEGSGAQGSGSAGEGSGAKGSGSAGEGSGAQGGSSAG-  122

PTP2c      -----------EGSGAKGSGSAGEGNAPEKGAEGSGQAQKPGIGQAGPGNAAAQGDSTGA  288
PTP2b      GSGAKGGSSAGEGSGALGSGSAGEGNAPEKGAEGSGQAQKPGIGQAGPGNAAAQGDSTGA  204
PTP2a      -----------EGSGAQGSGSAGEGNAPEKGAEGSGQAQKPGIGQAGPGNAAAQGDSTGA  171

PTP2c      ANDLVALETHKALKTALNQSMANSVSPQTANEFLAGNPECNQQAVEGNWKVINEKIKQEC  348
PTP2b      ANDLVALETHKALKTALNQSMANSVSPQTANEFLAGNPECNQQAVEGNWKVINEKIKQEC  264
PTP2a      ANDLVALETHKALKTALNQSMANSVSPQTANEFLAGNPECNQQAVEGNWKVINEKIKQEC  231

PTP2c      RNKMNERKAKAEQIAKFITKPTPDKCVKKLNPNALVCQALTVMNTIIKQPRFKIDLYGSN  408
PTP2b      RNKVNERKAKAEQIAKFITKPTPDKCVKKLNPNALVCQALTVMNTIIKQPRFKIDLYGSN  324
PTP2a      RNKVNERKAKAEQIAKFITKPTPDKCVKKLNPNALVCQALTVMNTIIKQPRFKIDLYGSN  291

PTP2c      VVEIKKDGSPILLGVAESLNYNVTKMKRKRYNPLKNPTARLEFNELGSLLKQEGEIPKPK  468
PTP2b      VVEIKKDGSPILLGVAESLNYNVTKMKRKRYNPLKNPTARLEFNELGSLLKQEGEIPKPK  384
PTP2a      VVEIKKDGSPILLGVAESLNYNVTKMKRKRYNPLKNPTARLEFNELGSLLKQEGEIPKPK  351

PTP2c      RLDPCGSCLEKLSKKAQVEGEKDMCGGCDALMELNSLKRGSFNNLQTENKKPEVTPTETT  528
PTP2b      RLDPCGSCLEKLSKKAQVEGEKDMCGGCDALMELNSLKRGSFNNLQTENKKPEVTPTETT  444
PTP2a      RLDPCGSCLEKLSKKAQVEGEKDMCGGCDALMELNSLKRGSFNNLQTENKKPEVTPTETT  411

PTP2c      KAAEESK                                                       535
PTP2b      KAAEESK                                                       451
PTP2a      KAAEESK                                                       418
```
