# Supplementary material for: Identification and localization of polar tube proteins in the extruded polar tube of the microsporidian Anncaliia algerae
Source: Sci Rep. 2023 May 30;13:8773. doi: 10.1038/s41598-023-35511-y (PMC10229552; doi:10.1038/s41598-023-35511-y)
Supplement: Supplementary file 2 — Supplementary Figure S2. [file 41598_2023_35511_MOESM2_ESM.pptx]

## Slide 1
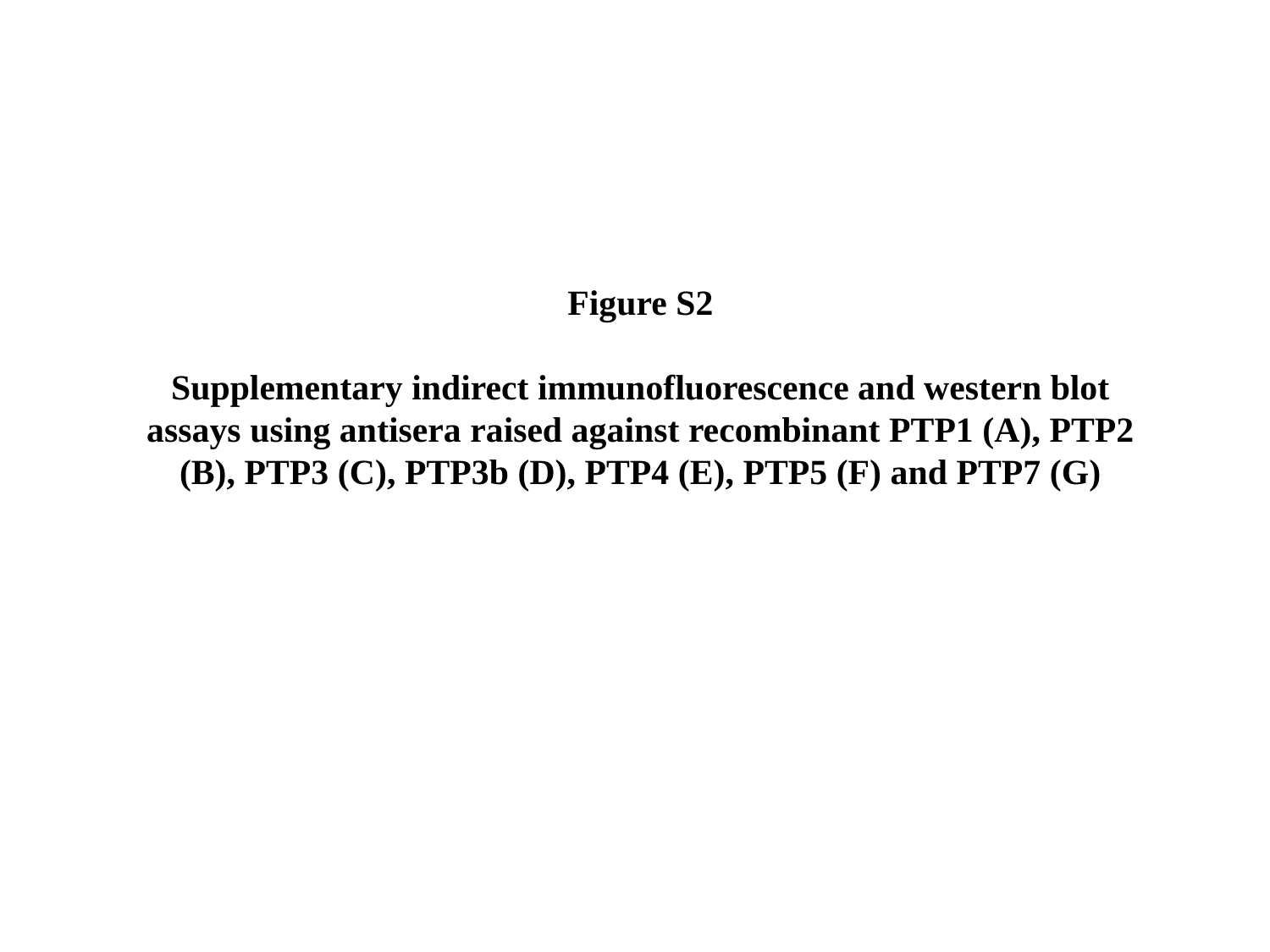

Figure S2
Supplementary indirect immunofluorescence and western blot assays using antisera raised against recombinant PTP1 (A), PTP2 (B), PTP3 (C), PTP3b (D), PTP4 (E), PTP5 (F) and PTP7 (G)

## Slide 2
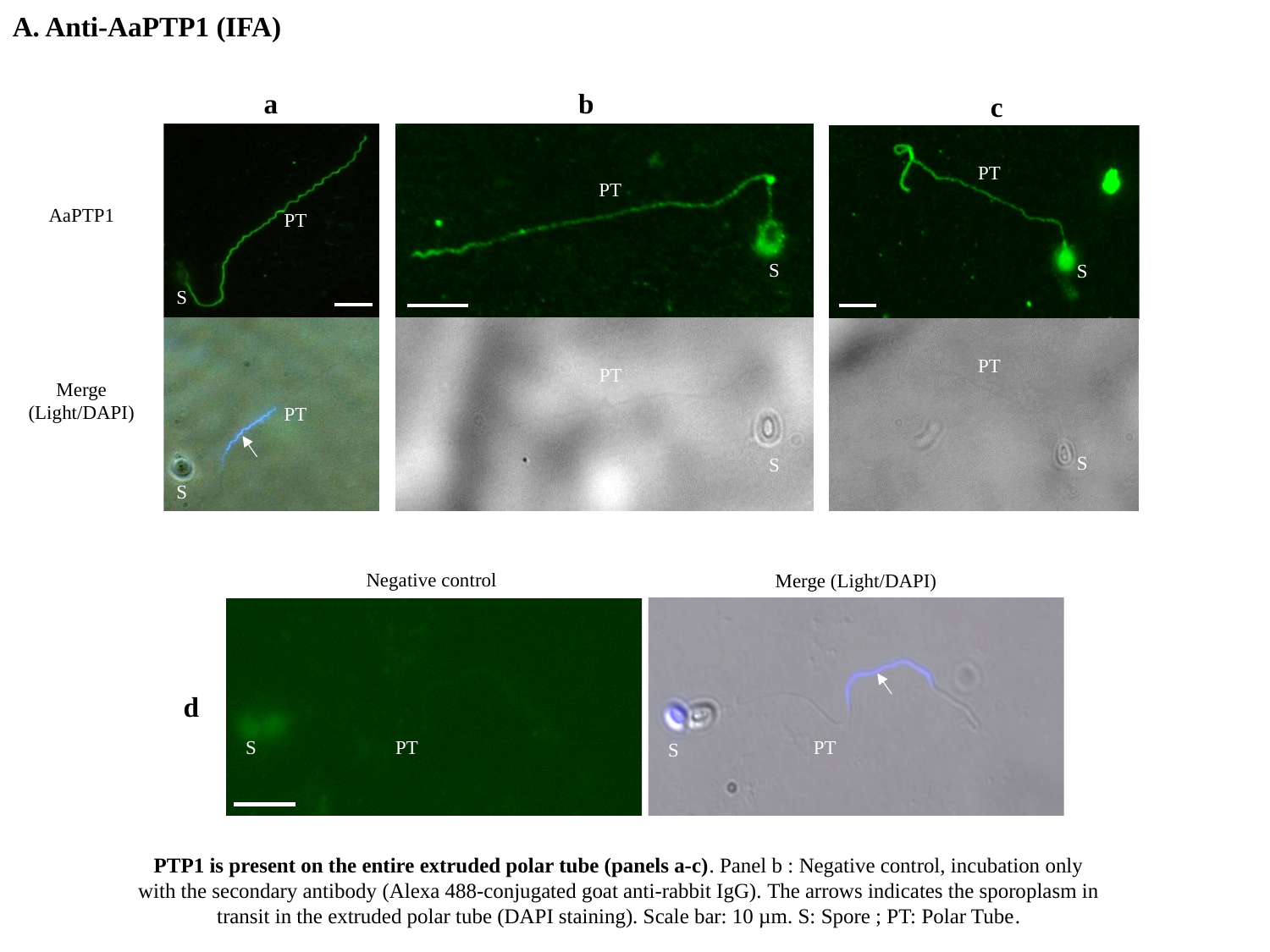

A. Anti-AaPTP1 (IFA)
a
b
c
PT
PT
AaPTP1
PT
S
S
S
PT
PT
Merge
(Light/DAPI)
PT
S
S
S
Negative control
Merge (Light/DAPI)
d
PT
S
PT
PT
S
S
PTP1 is present on the entire extruded polar tube (panels a-c). Panel b : Negative control, incubation only with the secondary antibody (Alexa 488-conjugated goat anti-rabbit IgG). The arrows indicates the sporoplasm in transit in the extruded polar tube (DAPI staining). Scale bar: 10 µm. S: Spore ; PT: Polar Tube.

## Slide 3
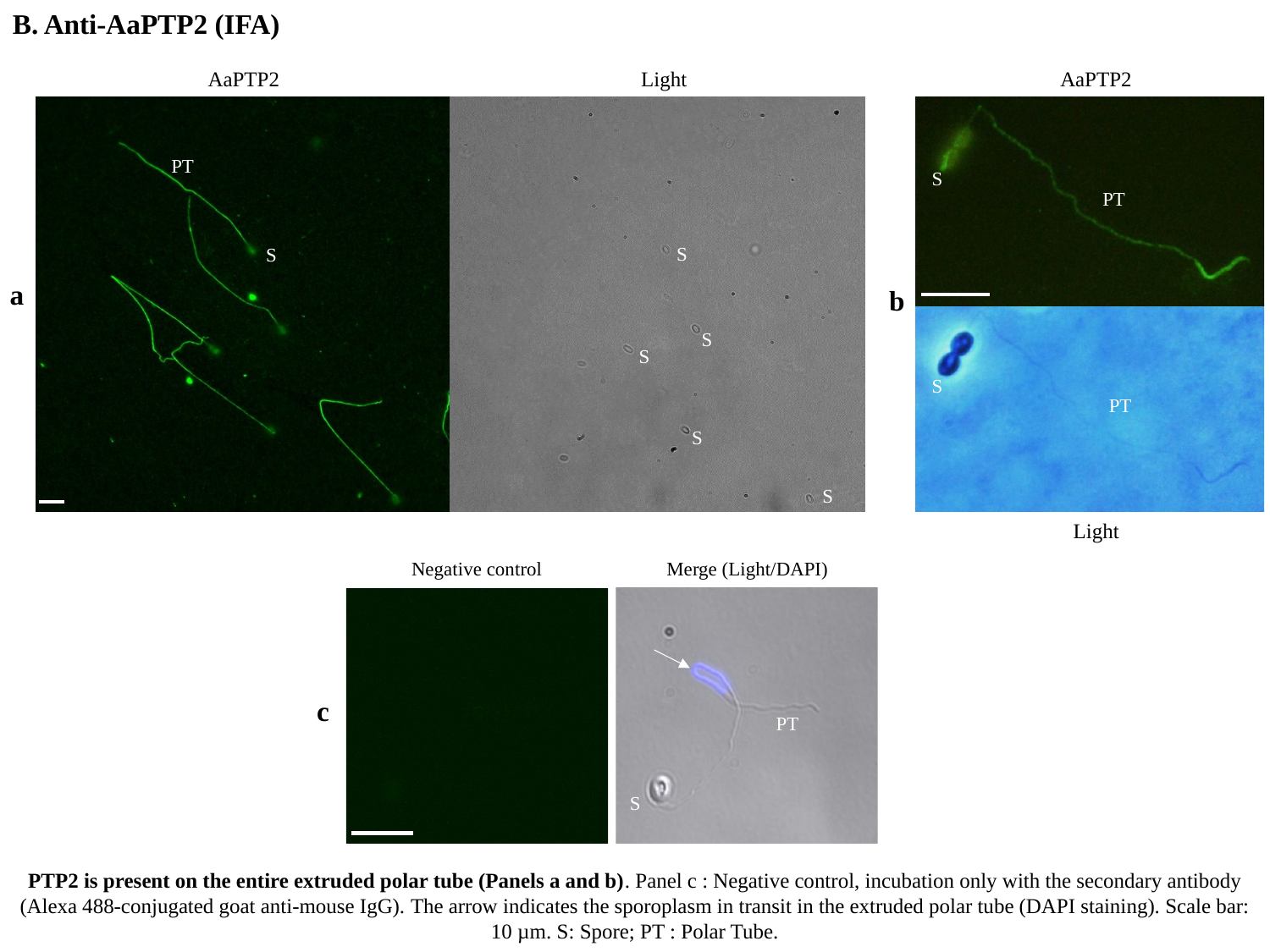

B. Anti-AaPTP2 (IFA)
AaPTP2
Light
AaPTP2
PT
S
PT
S
S
a
b
S
S
S
PT
S
S
Light
Merge (Light/DAPI)
Negative control
c
PT
S
PTP2 is present on the entire extruded polar tube (Panels a and b). Panel c : Negative control, incubation only with the secondary antibody (Alexa 488-conjugated goat anti-mouse IgG). The arrow indicates the sporoplasm in transit in the extruded polar tube (DAPI staining). Scale bar: 10 µm. S: Spore; PT : Polar Tube.

## Slide 4
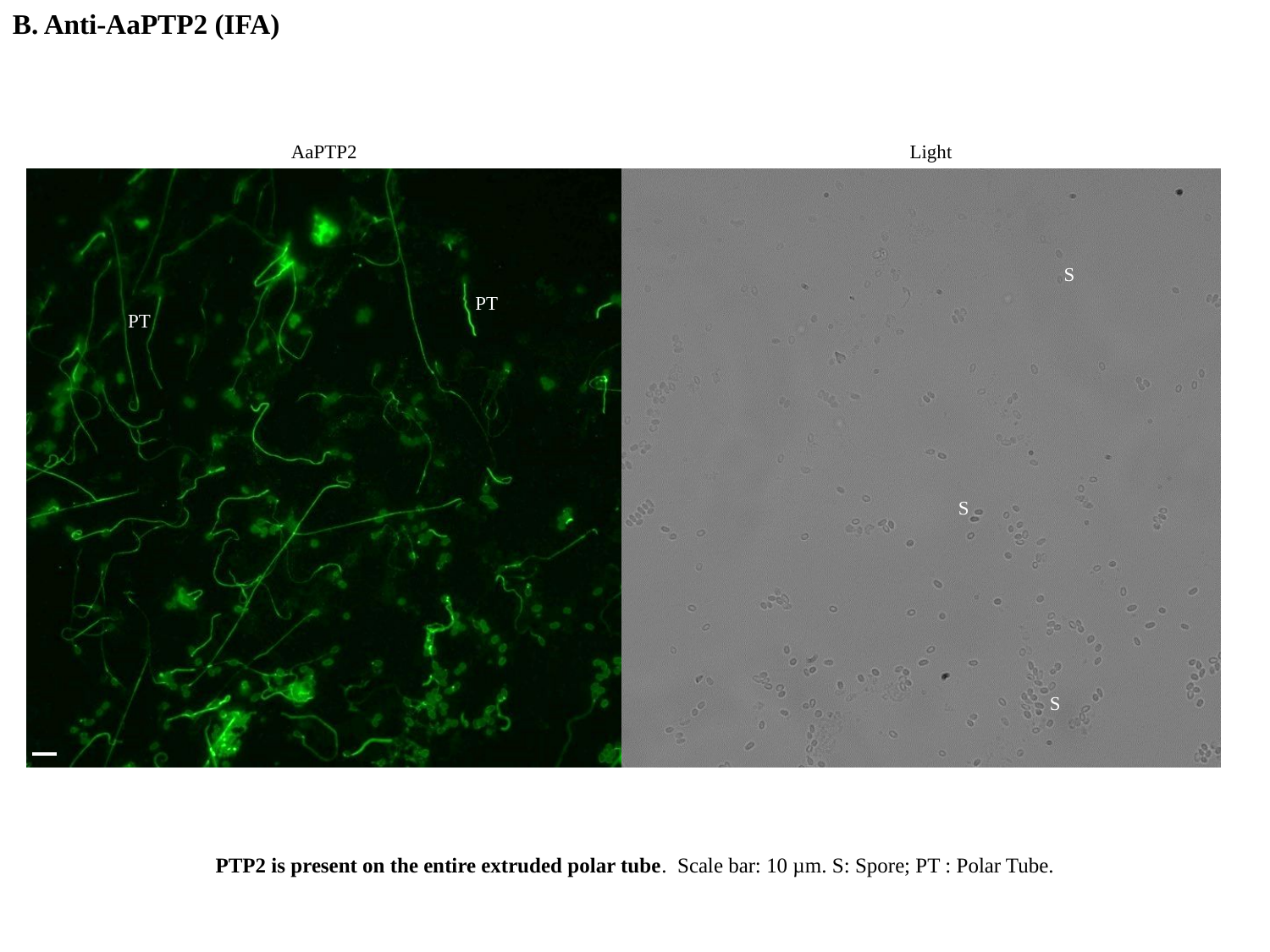

B. Anti-AaPTP2 (IFA)
AaPTP2
Light
S
PT
PT
S
S
PTP2 is present on the entire extruded polar tube. Scale bar: 10 µm. S: Spore; PT : Polar Tube.

## Slide 5
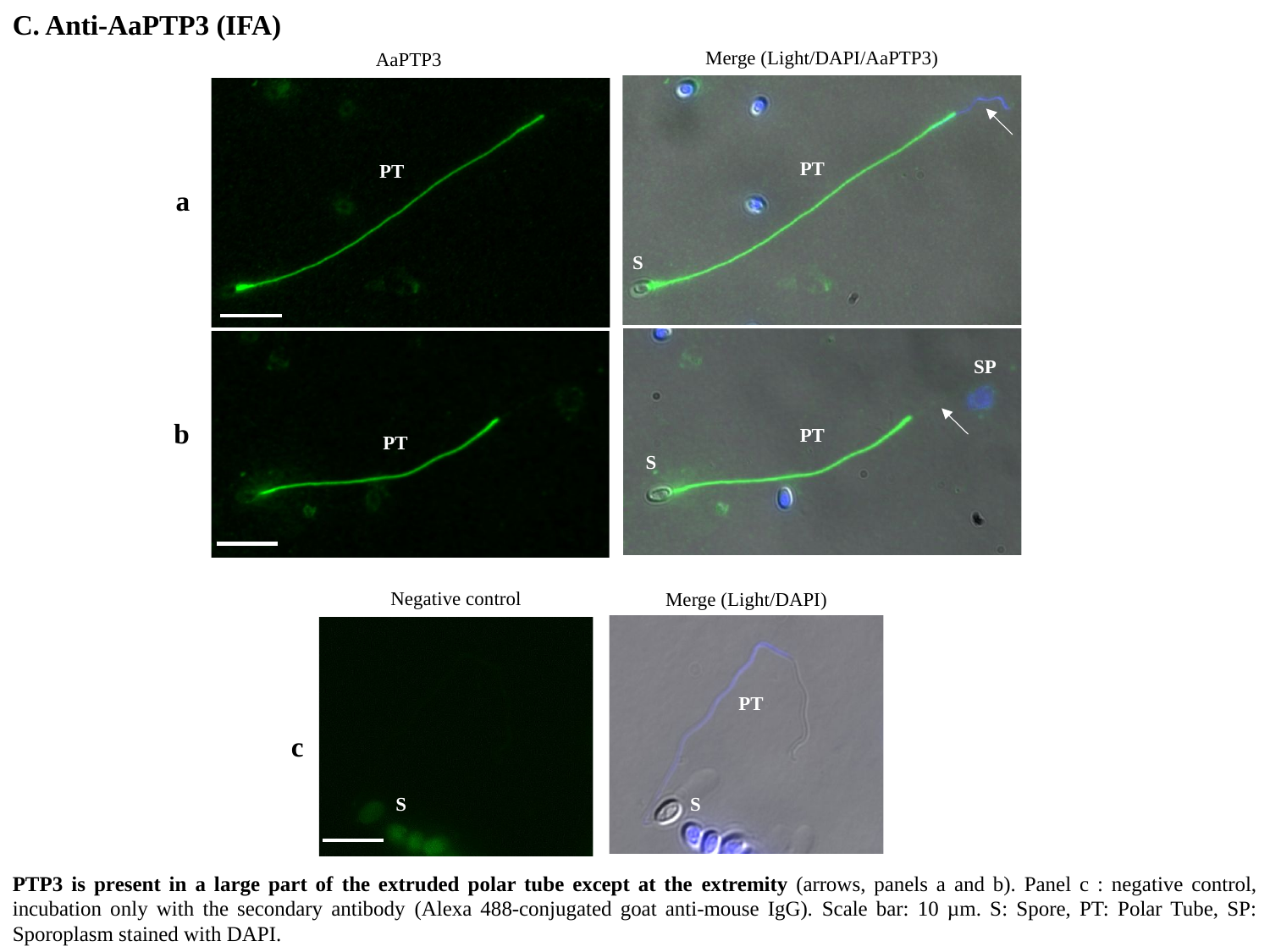

C. Anti-AaPTP3 (IFA)
Merge (Light/DAPI/AaPTP3)
AaPTP3
PT
PT
a
S
SP
b
PT
PT
S
Negative control
Merge (Light/DAPI)
PT
c
S
S
PTP3 is present in a large part of the extruded polar tube except at the extremity (arrows, panels a and b). Panel c : negative control, incubation only with the secondary antibody (Alexa 488-conjugated goat anti-mouse IgG). Scale bar: 10 µm. S: Spore, PT: Polar Tube, SP: Sporoplasm stained with DAPI.

## Slide 6
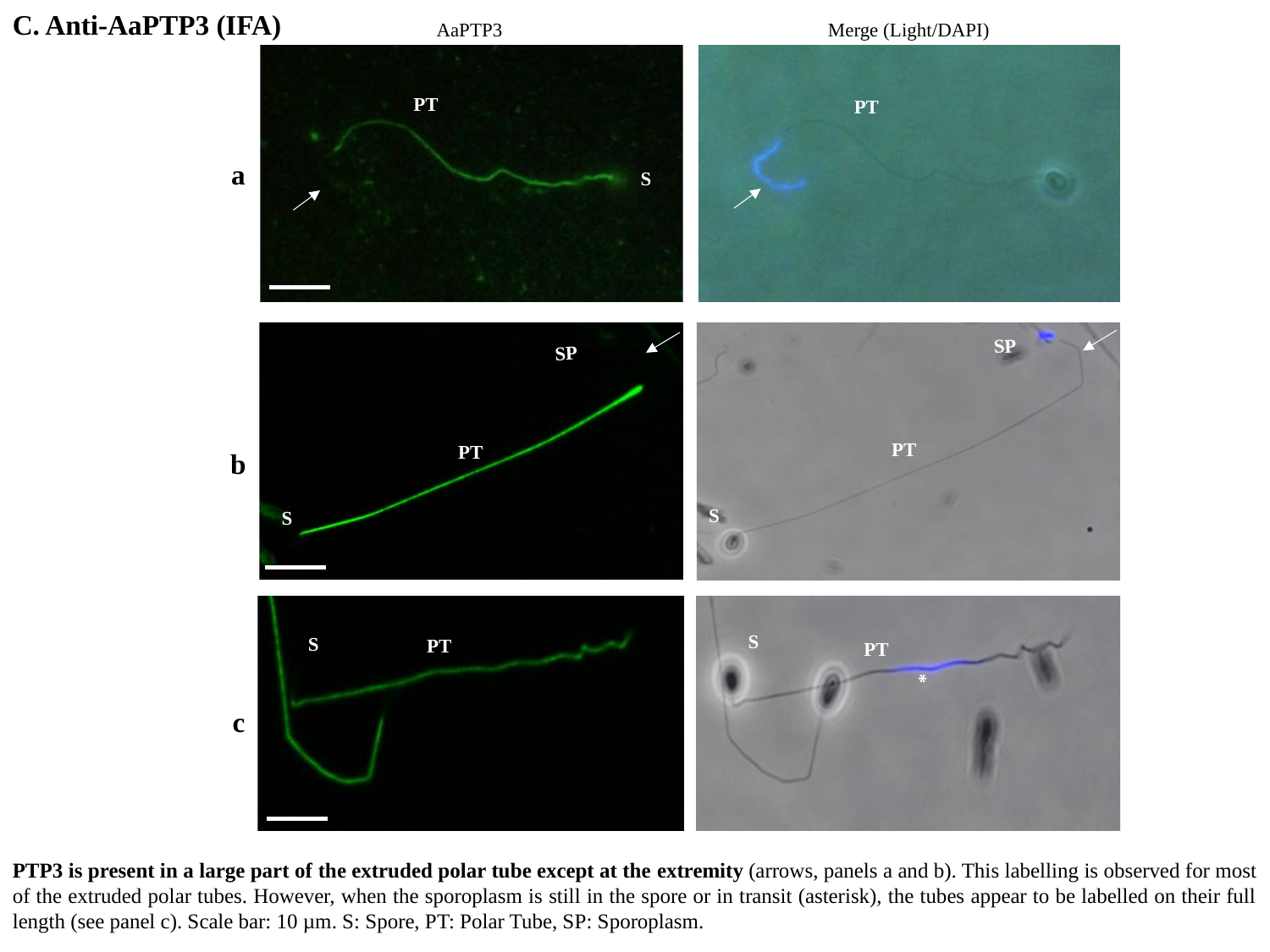

C. Anti-AaPTP3 (IFA)
Merge (Light/DAPI)
AaPTP3
PT
S
PT
a
S
SP
PT
S
SP
PT
b
S
*
S
S
PT
PT
c
PTP3 is present in a large part of the extruded polar tube except at the extremity (arrows, panels a and b). This labelling is observed for most of the extruded polar tubes. However, when the sporoplasm is still in the spore or in transit (asterisk), the tubes appear to be labelled on their full length (see panel c). Scale bar: 10 µm. S: Spore, PT: Polar Tube, SP: Sporoplasm.

## Slide 7
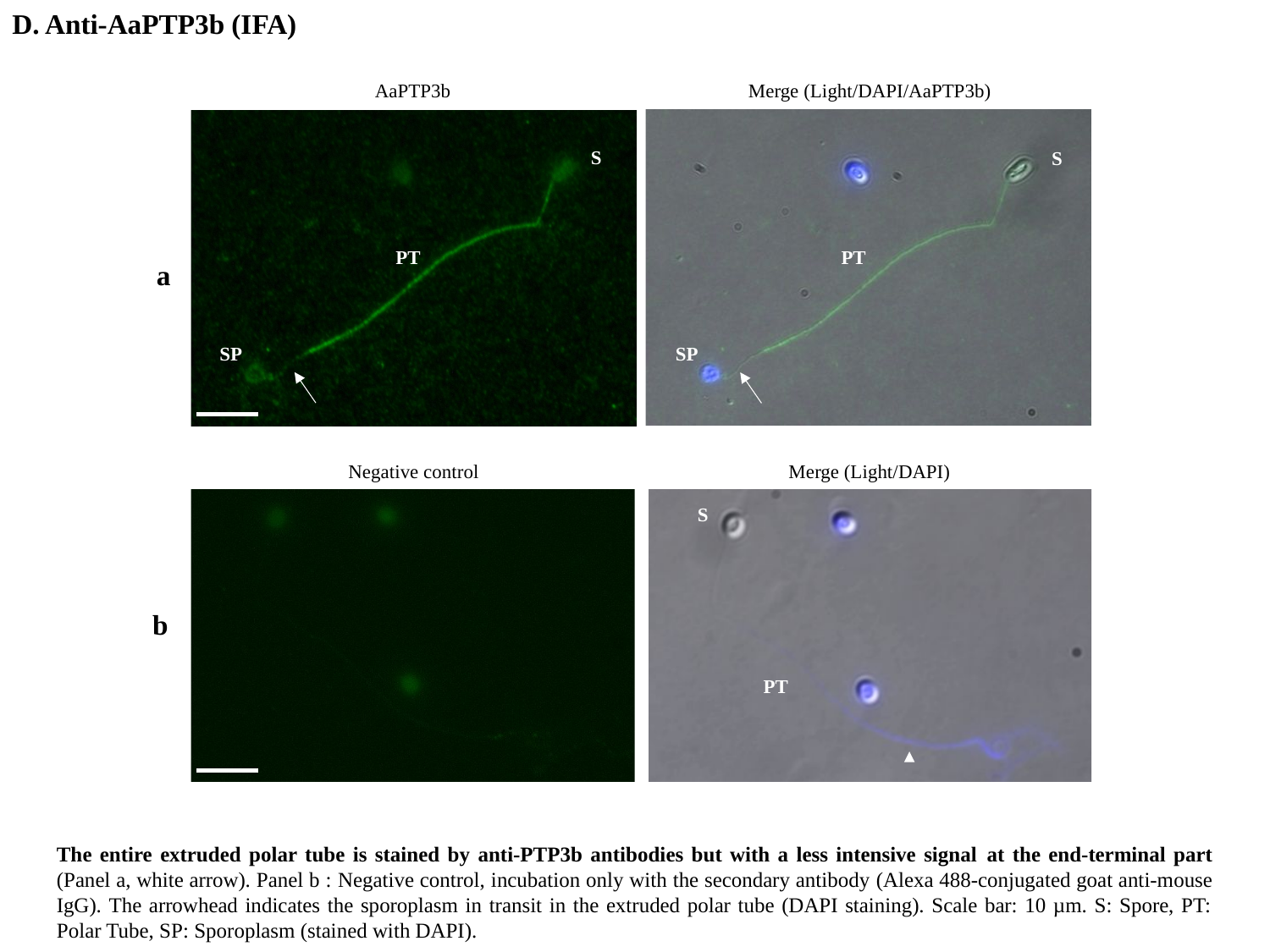

D. Anti-AaPTP3b (IFA)
Merge (Light/DAPI/AaPTP3b)
AaPTP3b
S
S
PT
PT
a
SP
SP
Negative control
Merge (Light/DAPI)
S
b
PT
The entire extruded polar tube is stained by anti-PTP3b antibodies but with a less intensive signal at the end-terminal part (Panel a, white arrow). Panel b : Negative control, incubation only with the secondary antibody (Alexa 488-conjugated goat anti-mouse IgG). The arrowhead indicates the sporoplasm in transit in the extruded polar tube (DAPI staining). Scale bar: 10 µm. S: Spore, PT: Polar Tube, SP: Sporoplasm (stained with DAPI).

## Slide 8
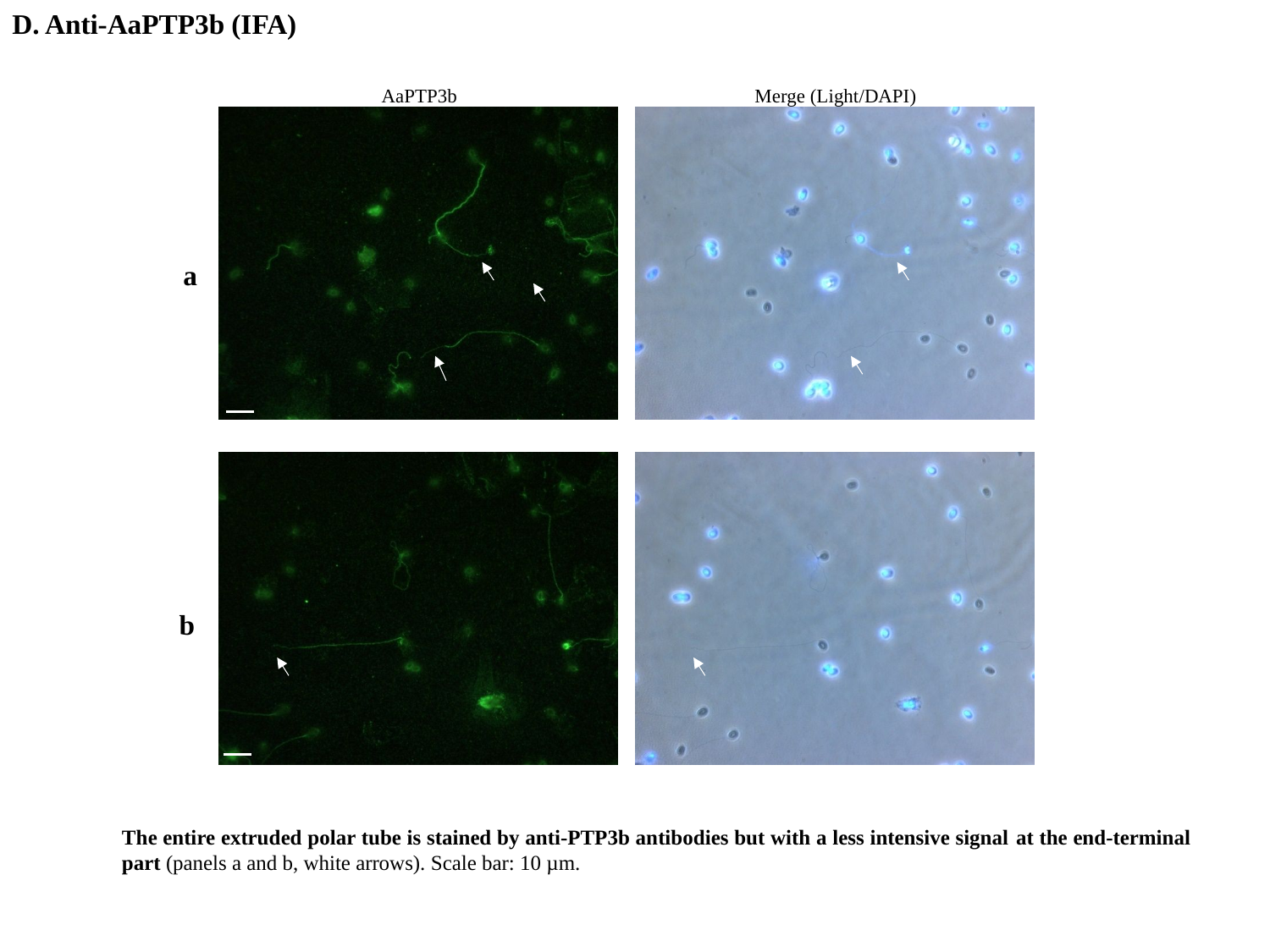

D. Anti-AaPTP3b (IFA)
AaPTP3b
Merge (Light/DAPI)
a
b
The entire extruded polar tube is stained by anti-PTP3b antibodies but with a less intensive signal at the end-terminal part (panels a and b, white arrows). Scale bar: 10 µm.

## Slide 9
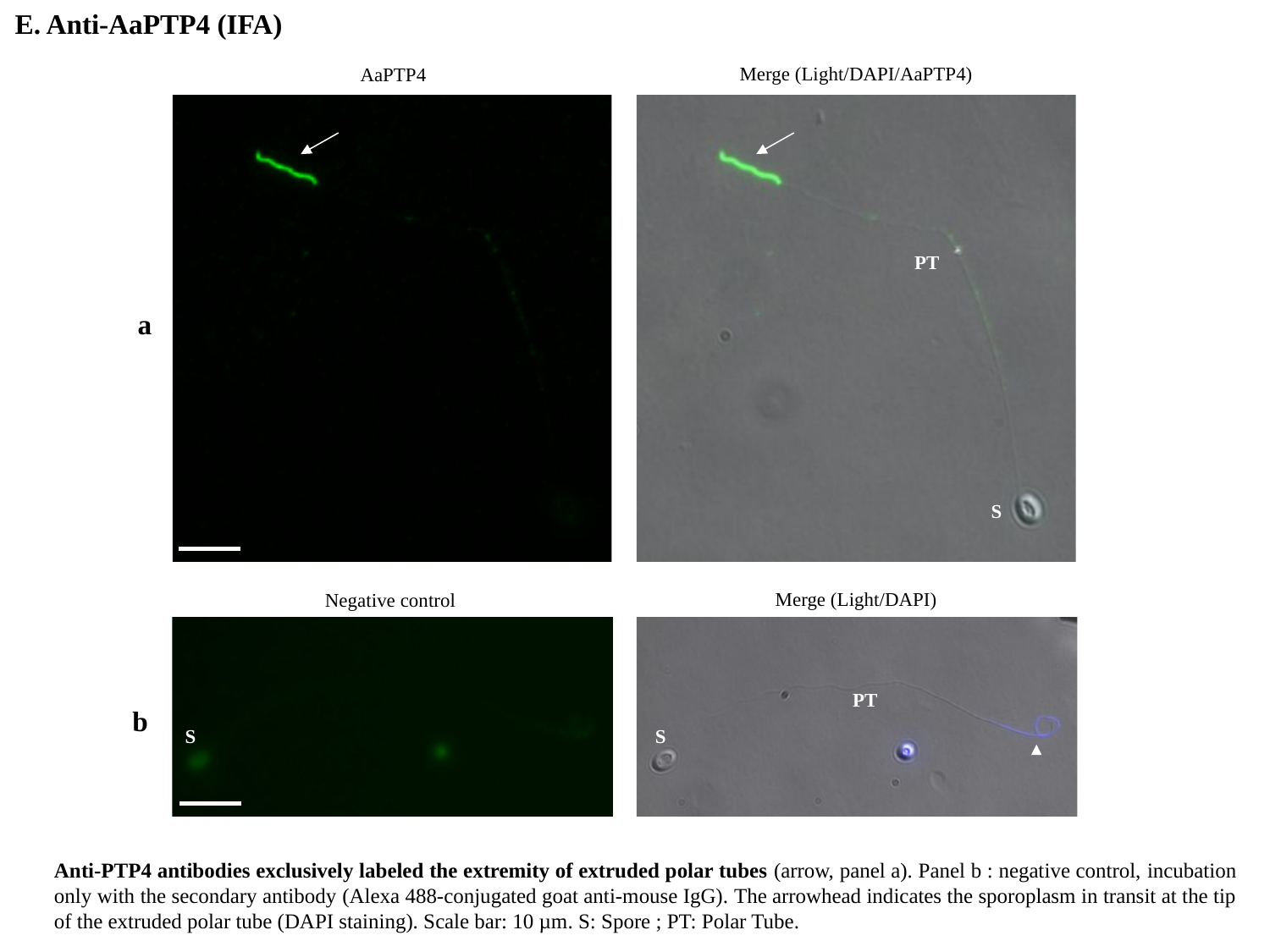

E. Anti-AaPTP4 (IFA)
Merge (Light/DAPI/AaPTP4)
AaPTP4
PT
a
S
Merge (Light/DAPI)
Negative control
PT
b
S
S
Anti-PTP4 antibodies exclusively labeled the extremity of extruded polar tubes (arrow, panel a). Panel b : negative control, incubation only with the secondary antibody (Alexa 488-conjugated goat anti-mouse IgG). The arrowhead indicates the sporoplasm in transit at the tip of the extruded polar tube (DAPI staining). Scale bar: 10 µm. S: Spore ; PT: Polar Tube.

## Slide 10
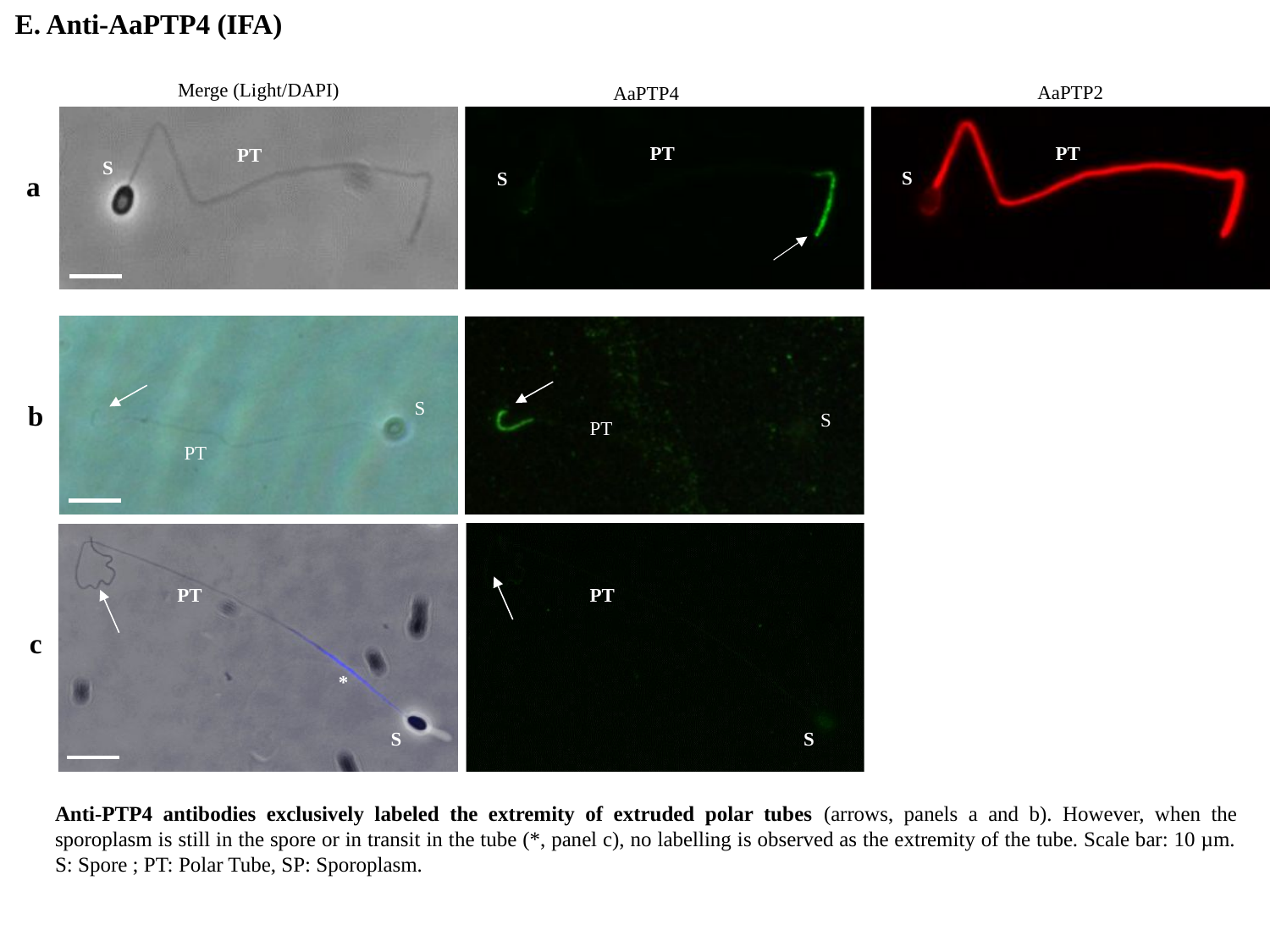

E. Anti-AaPTP4 (IFA)
Merge (Light/DAPI)
AaPTP2
AaPTP4
PT
PT
PT
S
S
S
a
S
b
S
PT
PT
PT
PT
c
*
S
S
Anti-PTP4 antibodies exclusively labeled the extremity of extruded polar tubes (arrows, panels a and b). However, when the sporoplasm is still in the spore or in transit in the tube (*, panel c), no labelling is observed as the extremity of the tube. Scale bar: 10 µm. S: Spore ; PT: Polar Tube, SP: Sporoplasm.
Echelles : 10 µm

## Slide 11
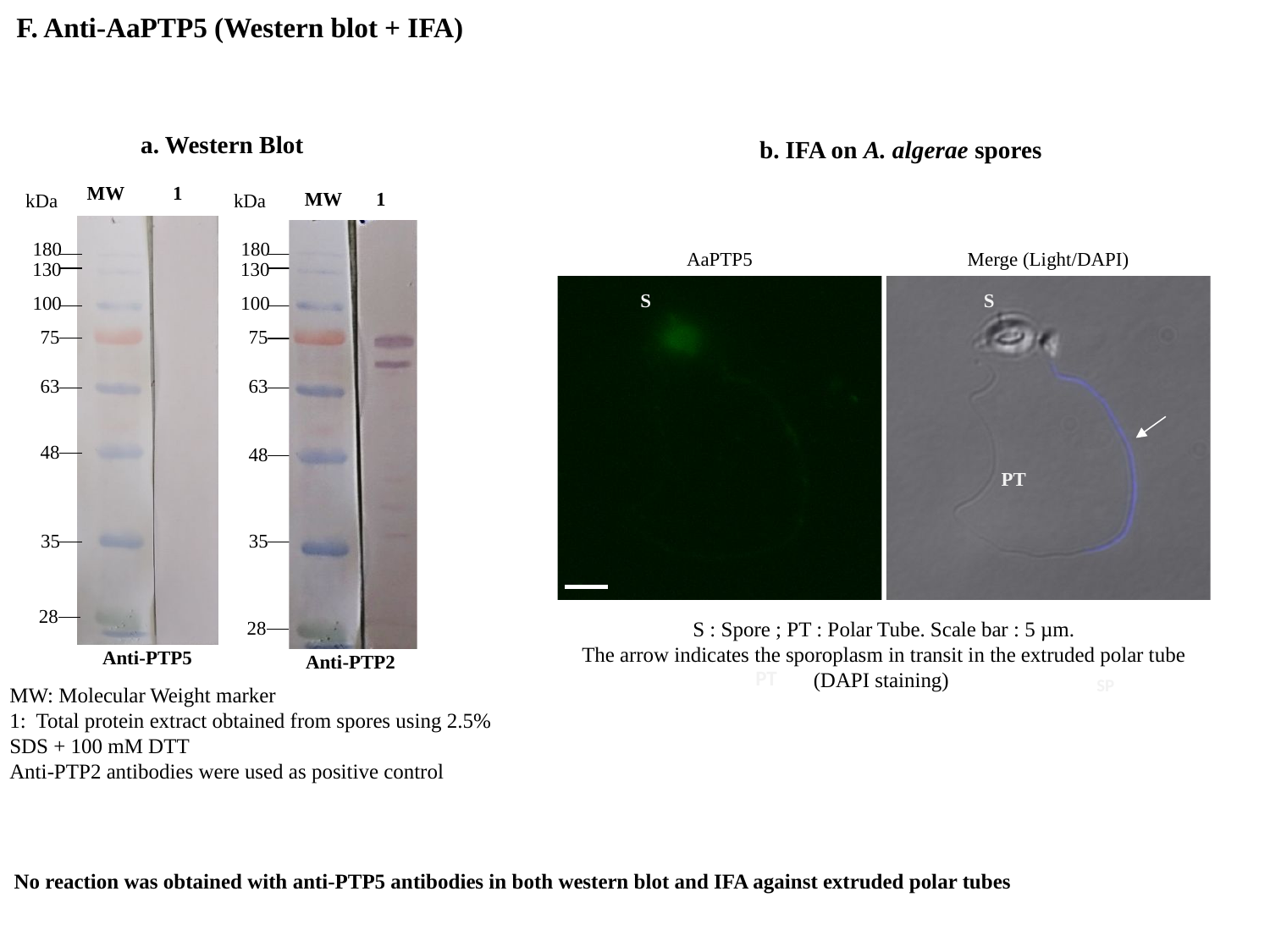

F. Anti-AaPTP5 (Western blot + IFA)
a. Western Blot
b. IFA on A. algerae spores
MW 1
MW 1
kDa
kDa
180
180
AaPTP5
Merge (Light/DAPI)
130
130
S
S
100
100
75
75
63
63
48
48
PT
S
35
35
28
28
S : Spore ; PT : Polar Tube. Scale bar : 5 µm.
The arrow indicates the sporoplasm in transit in the extruded polar tube (DAPI staining)
Anti-PTP5
Anti-PTP2
PT
SP
MW: Molecular Weight marker
1: Total protein extract obtained from spores using 2.5% SDS + 100 mM DTT
Anti-PTP2 antibodies were used as positive control
No reaction was obtained with anti-PTP5 antibodies in both western blot and IFA against extruded polar tubes

## Slide 12
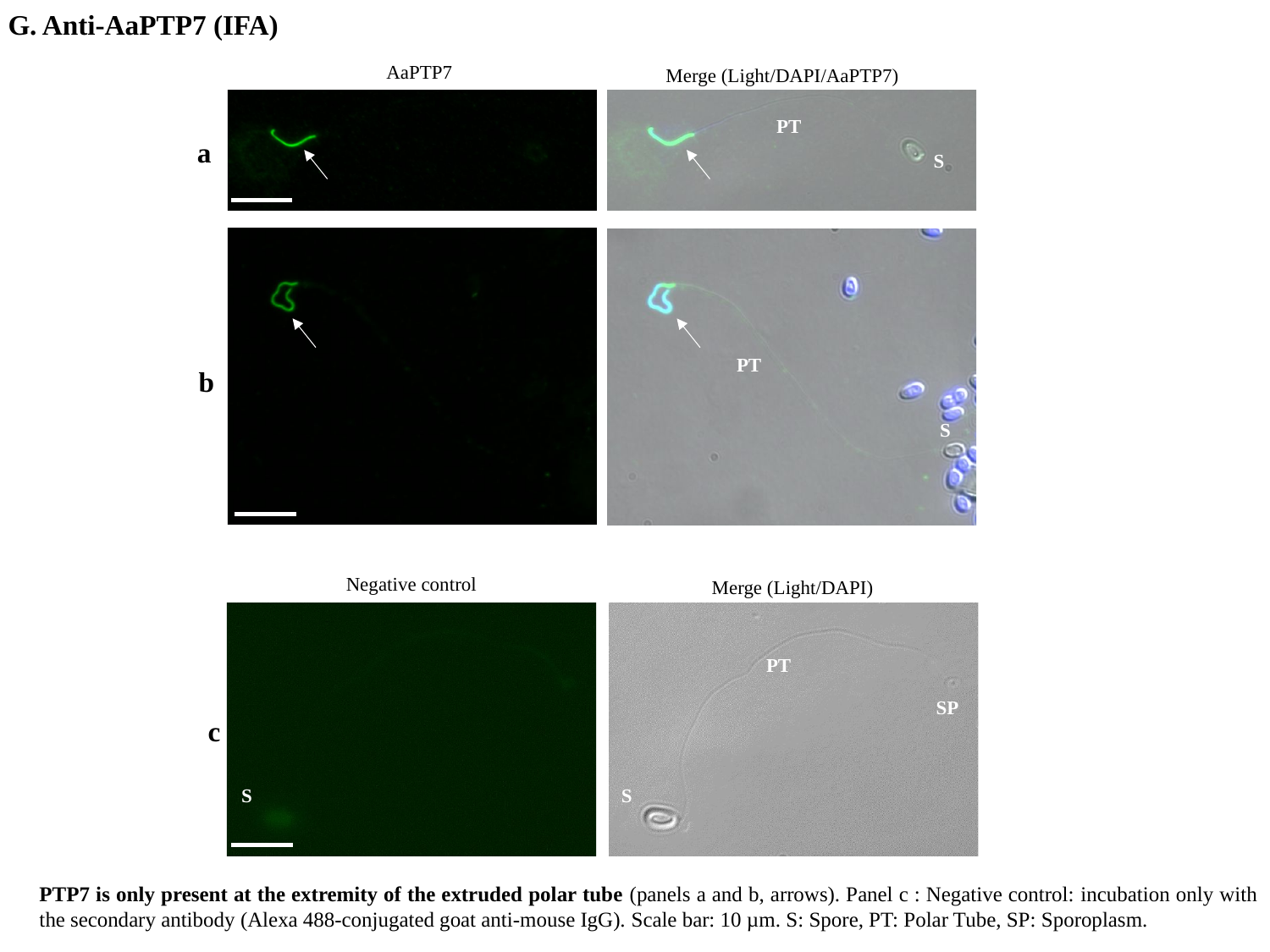

G. Anti-AaPTP7 (IFA)
AaPTP7
Merge (Light/DAPI/AaPTP7)
PT
a
S
PT
b
S
Negative control
Merge (Light/DAPI)
PT
SP
c
S
S
PTP7 is only present at the extremity of the extruded polar tube (panels a and b, arrows). Panel c : Negative control: incubation only with the secondary antibody (Alexa 488-conjugated goat anti-mouse IgG). Scale bar: 10 µm. S: Spore, PT: Polar Tube, SP: Sporoplasm.

## Slide 13
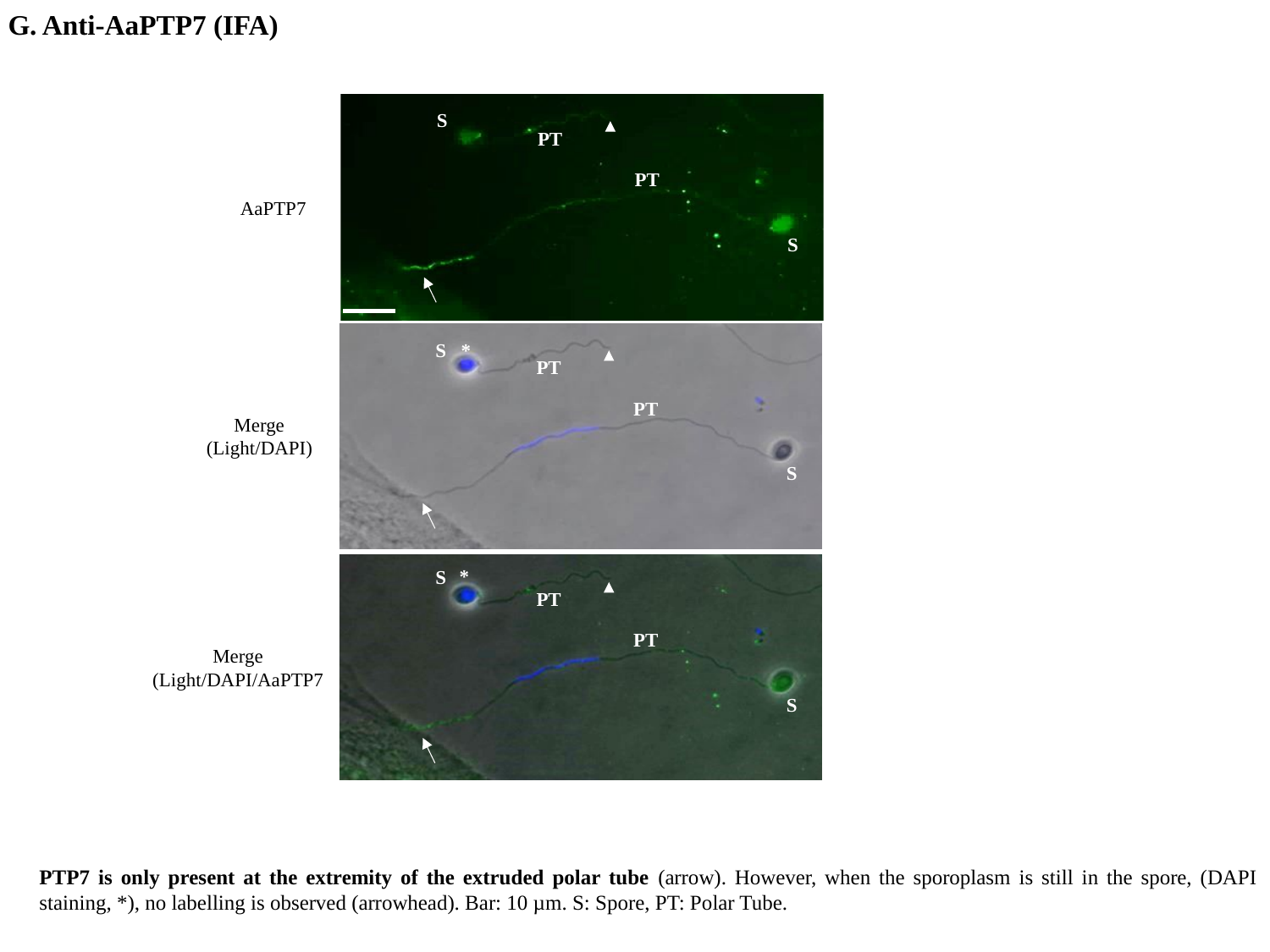

G. Anti-AaPTP7 (IFA)
S
PT
PT
AaPTP7
b
S
a
S
*
a
PT
PT
Merge (Light/DAPI)
S
c
*
S
PT
PT
Merge (Light/DAPI/AaPTP7
S
PTP7 is only present at the extremity of the extruded polar tube (arrow). However, when the sporoplasm is still in the spore, (DAPI staining, *), no labelling is observed (arrowhead). Bar: 10 µm. S: Spore, PT: Polar Tube.
