# Supplementary figures and images for: Identification and localization of polar tube proteins in the extruded polar tube of the microsporidian Anncaliia algerae
Source: Sci Rep. 2023 May 30;13:8773. doi: 10.1038/s41598-023-35511-y (PMC10229552; doi:10.1038/s41598-023-35511-y)

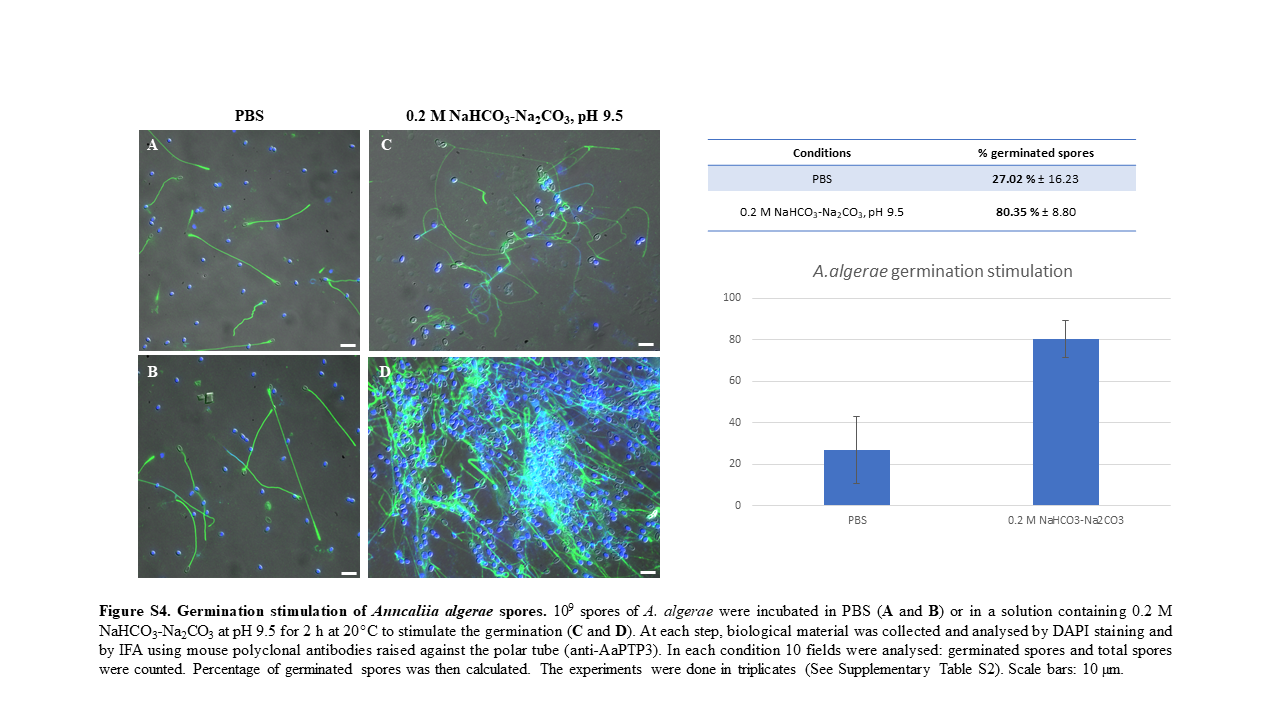

Supplement: Supplementary file 4 — Supplementary Figure S4. [file 41598_2023_35511_MOESM4_ESM.tif]
