# Supplementary material for: Identification and localization of polar tube proteins in the extruded polar tube of the microsporidian Anncaliia algerae
Source: Sci Rep. 2023 May 30;13:8773. doi: 10.1038/s41598-023-35511-y (PMC10229552; doi:10.1038/s41598-023-35511-y)
Supplement: Supplementary file 5 — Supplementary Table S1. [file 41598_2023_35511_MOESM5_ESM.docx]

**Table S1 Nucleotide sequences of primers used for pGEX 4T-1 cloning to produce recombinant proteins**. Amplified regions were arbitrarily chosen apart from the signal peptide. ^a^ Number of amino acids and size of the recombinant protein include the Glutathion S-Transferase (GST), the PTP and the histidine tag. Three different constructions were done for PTP1 (full length, N terminal part and C terminal part) and two for PTP2 (variable glycine rich N-terminal part and the lysine rich C-terminal region). bp: base pair ; aa: amino acids ; kDa: kilodaltons. *Ptps* genes accession numbers are respectively: OM728538 (*ptp1*), OM728539 (*ptp2*), OM728540 (*ptp3*), OM728541 (*ptp3b*), OM728542 (*ptp4*), OM728543 (*ptp5*) and OM728544 (*ptp7*).

| **Genes** | **Gene size** | **Number of amino acids of the complete protein** | **Primers** | **Amplified fragment size** | **Corresponding region in amino acids** | **Recombinant protein size^a^** |
| --- | --- | --- | --- | --- | --- | --- |
| *ptp1* | 1224 bp | 407 aa | Aa_PTP1_Bam  5’-ATGGGATCCGCAAAGGCAATTCAAGCTGAG-3’ | 1185 bp | 18 - 407 | 625 aa  (66.8 kDa) |
|  |  |  | Aa_PTP1_Eco  5’-GAATTCACATGTGCATCCTCCATTAG-3’ |  |  |  |
|  |  |  | Aa_PTP1_Nterm_Bam  5’- GGGGGATCCGCAAAGGCAATTCAAGCTGAG-3’ | 447 bp | 18 - 162 | 390 aa  (43.5 kDa) |
|  |  |  | Aa_PTP1_Nterm_Eco  5’- GGGGAATTCTGGAACAGGTACCTCCTCAC-3’ |  |  |  |
|  |  |  | Aa_PTP1_Cterm_Bam  5’- GGGGGATCCACGGCACCACCAACATCACC-3’ | 531 bp | 235 - 407 | 418 aa  (45.9 kDa) |
|  |  |  | Aa_PTP1_Cterm_Eco  5’- GGGGAATTCACATGTGCATCCTCCATTAG-3’ |  |  |  |
| *ptp2* | 1608 bp | 535 aa | Aa_PTP2N_EcoRI  5’- GGGGAATTCATGTCGGGAGTTAGCTCTGGC-3’ | 630 bp | 53 - 262 | 454 aa  (44.5 kDa) |
|  |  |  | Aa_PTP2N_XhoI  5’- GGGCTCGAGGCCTTCTGCGCCTTTCTCAGG-3’ |  |  |  |
|  |  |  | Aa_PTP2F  5’-GCCAAAGCTGAACAAATAGC-3‘ | 531 bp | 406 - 533 | 367 aa  (42 kDa) |
|  |  |  | Aa_PTP2_Xho  5’-GGGCTCGAGCTCCTCTGCTGCTTTAGTAG-3’ |  |  |  |
| *ptp3* | 3609 bp | 1203 aa | Aa_PTP3_Bam  5’-GGGGGATCCGGTCATATGCAATTGGAAGG-3‘ | 618 bp | 181 - 380 | 447 aa  (49.1 kDa) |
|  |  |  | Aa_PTP3_Eco  5’-GGGGAATTCATTTCCAGCCATGATTGCTC-3‘ |  |  |  |
| *ptp3b* | 3594 bp | 1197 aa | Aa_PTP3b_Bam  5’-GGGGGATCCCCCGATGATGCAAAAGAGG-3‘ | 750 bp | 681 - 924 | 489 aa  (55.3 kDa) |
|  |  |  | Aa_PTP3b_Eco  5’-GGGGAATTCACCAGAGCCGTTAGGAGGC-3‘ |  |  |  |
| *ptp4* | 765 bp | 254 aa | Aa_PTP4_Bam  5’-ATGGGATCCATGCTGTGTACCTCTCAGG-3’ | 705 bp | 10 - 239 | 475 aa  (54.8 kDa) |
|  |  |  | Aa_PTP4_Eco  5’-GAATTCGCTTCCTTCATAATTAGATTC-3’ |  |  |  |
| *ptp5* | 723 bp | 240 aa | Aa_PTP5_Bam  5‘-ATGGGATCCGCCAGAAGATTGGATTAC-3’ | 630 bp | 36 - 240 | 450 aa  (52.1 kDa) |
|  |  |  | Aa_PTP5_Eco  5‘-GAATTCTGATGGACAAATGAAATCAAG-3’ |  |  |  |
| *ptp7* | 1254 bp | 417 aa | Aa_PTP7_Bam  5’- GGGGGATCCGGAACTAACGGGAATAGG-3’ | 223 bp | 28 - 132 | 350 aa  (40.4 kDa) |
|  |  |  | Aa_PTP7_Eco  5’- GGGGAATTCTCTTTGGGCTTTGACTCC-3’ |  |  |  |
